# Correlation of clinical features and genetic profiles of stromal interaction molecule 1 (*STIM1*) in colorectal cancers

**DOI:** 10.18632/oncotarget.5888

**Published:** 2015-11-03

**Authors:** Henry Sung-Ching Wong, Wei-Chiao Chang

**Affiliations:** ^1^ Master Program for Clinical Pharmacogenomics and Pharmacoproteomics, School of Pharmacy, Taipei Medical University, Taipei, Taiwan; ^2^ Department of Clinical Pharmacy, School of Pharmacy, Taipei Medical University, Taipei, Taiwan; ^3^ Department of Pharmacy, Taipei Medical University Wan Fang Hospital, Taipei, Taiwan; ^4^ Center for Biomarkers and Biotech Drugs, Kaohsiung Medical University, Kaohsiung, Taiwan; ^5^ Department of Pharmacy, Taipei Medical University Wan Fang Hospital, Taipei, Taiwan

**Keywords:** colorectal cancer, stored-operated calcium entry pathway, stromal interaction molecule 1, bioinformatics, data mining

## Abstract

*STIM1* overexpression has been observed in a portion of colorectal cancer (CRC) patients and associated with cancer cell invasion and migration. To characterize the distinctive expression profiles associated with stromal interaction molecule 1 (*STIM1)* overexpression/low-expression between CRC subtypes, and further assess the divergence transcription regulation impact of *STIM1* between colon (COADs) and rectum (READs) adenocarcinomas in order to depict the role of SOCE pathway in CRCs, we have conducted a comprehensive phenome-transcriptome-interactome analysis to clarify underlying molecular differences of COADs/READs contributed by *STIM1*. Results demonstrated that a number of novel *STIM1*-associated signatures have been identified in COADs but not READs. Specifically, the presence of *STIM1* overexpression in COADs, which represented a disturbance of the SOCE pathway, was associated with cell migration and cell motility properties. We identified 11 prognostic mRNA/miRNA predictors associated with the overall survival of COAD patients, suggesting the correlation of *STIM1*-associated features to clinicopathological outcomes. These findings enhance our understanding on differences between CRC subtypes in panoramic view, and suggested *STIM1* as a promising therapeutic biomarker in COADs.

## INTRODUCTION

Colorectal cancer (CRC) is the fourth most frequent human malignancy in men and is the third most frequent in women worldwide, as well as the second most common cause of cancer-related morbidity and mortality [1]. CRC transformation from the normal colonic mucosa arises through a progressive accumulation of genetic and epigenetic changes. In CRC, colon adenocarcinomas (COADs) and rectal adenocarcinomas (READs) are largely anatomically diverse CRC classifications. However, the correlation between molecular profiles and anatomic-classified COADs/READs is inconspicuous. In addition, epidemiological evidence revealed that colon and rectal carcinomas differ in terms of their prognoses, with colon cancer showing a greater aggressiveness and poorer clinical outcomes than rectal cancer.

The invasion-metastasis cascade involves sequential steps of local invasion, intravasation, transition, extravasation, and colonization which can be used to describe the distinctive nature of carcinomas [2]. Carcinomas at the invasive front undergo “the epithelial-mesenchymal transition (EMT)” which enables progression to occur, a process which involves multiple signaling pathway changes in CRC including transforming growth factor (TGF)-β signaling, WNT signaling, and so on. However, recent microarray profiling techniques have identified different expression features between colon and rectal carcinomas [3], and demonstrated substantial biological heterogeneity between colon and rectal carcinomas. Despite the identified pathway differences between colon and rectal carcinomas, the molecular or pathway root causes of cancer aggressiveness differ in colon/rectal carcinomas, and differences that underlie the prognosis potential remain obscure.

Recent studies have illustrated that Ca^2+^ signaling is increasingly implicated in CRC invasion and metastasis. The predominant Ca^2+^ signaling mechanism in most tumor cells is store-operated Ca^2+^ entry (SOCE), and SOCE-mediated Ca^2+^ oscillation is critical for focalized proteolysis, which is exploited by cancers to accelerate invasion and metastasis. The endoplasmic reticular (ER) Ca^2+^ sensor, stromal interaction molecule 1 (*STIM1*), which regulates the Ca^2+^ entry process in response to external stimuli, is thought to play a central role in coordinating Ca^2+^ signaling. It is known that *STIM1*-mediated Ca^2+^ oscillation controls invadopodium formation and focal adhesion turnover, and ultimately orchestrates tumor cell invasion and migration [4, 5]. In our previous study on CRC, STIM1 overexpression increased CRC aggressiveness, COX-2 gene activation and promoted tumor progression [6, 14]. In addition, blockage of the SOCE alleviated the aggressiveness of tumor cells. Those results suggested the importance of *STIM1* overexpression in the tumor invasion-metastasis cascade, and therefore *STIM1* may be developed as a potential therapeutic target of cancer treatment. Nevertheless, impacts of Ca^2+^ signaling and SOCE pathway aberrations on different CRC subtypes, i.e., colon and rectal carcinomas, remain exclusive. To address these issues, we conducted an integrated analysis focused on the transcriptome and interactome in colon and rectal carcinomas with distinctive invasiveness and aggressiveness features.

The Cancer Genome Atlas (TCGA) project focusing on CRC was carried out at 2012 [7], and data are available through its data portal. Using biological computational techniques, we comprehensively analyzed all available transcriptomic profiles (including messenger (m)RNA and micro (mi)RNA data) to gain a panoramic view of expression patterns between colon and rectal cancers. Furthermore, correlation analyses between tumor aggressiveness behaviors and expression patterns were carried out to point out the fundamental molecular contributions to the clinical tumor invasion status.

## RESULTS

### Correlation between patients' clinical profile and STIM1 expression values in CRC

**Figure 1:**
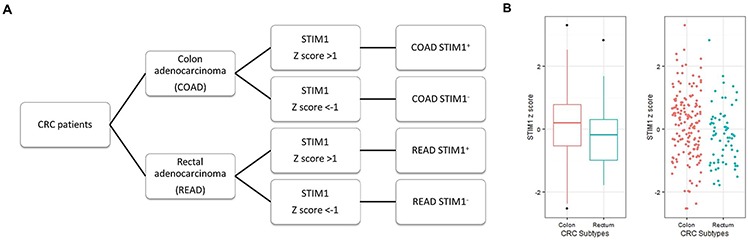
Patient categorization for *STIM1*-mediated Ca^2+^ signaling analysis based on colorectal cancer (CRC) subtypes and the *STIM1* expression status **A.** Patient selection criteria used for the following analysis (microarray, mRNA sequencing, and miRNA sequencing data). CRC patients were anatomically categorized into colon adenocarcinomas (COADs) and rectal adenocarcinomas (READs). These patients were further stratified into *STIM1*+ and *STIM1*- groups based on the presence or absence of *STIM1* overexpression. Patients with a *STIM1* z score of >+1 or <-1 were selected to undergo differentially expressed gene identification. **B.** A whisker boxplot and jitter plot summarizing differences in normalized *STIM1* expression levels (Z scores) among COADs and READs (Wilcoxon Mann-Whitney rank sum *p* = 0.0066).

CRC patients with available *STIM1* expression data were separated into COAD (*n* = 154) and READ (*n* = 68) groups (Figure 1A). In each CRC subtype, correlation analyses between *STIM1* expression values and clinical profiles were conducted. First, we assessed the *STIM1* expression value between COAD and READ patients, and the expression value of *STIM1* was significantly higher in COAD (mean ± SD = 0.144 ± 1.065) than READ (mean ± SD = −0.202 ± 0.906) patients (exact Wilcoxon Mann-Whitney rank sum *p* = 0.0066, Figure 1B). In the dichotomized *STIM1* group, 27 of 154 (17.5%) were *STIM1* overexpression and 20 of 154 (13.0%) were *STIM1* low-expression in COADs. In READs, only 5 of 68 (7.4%) were *STIM1* overexpression and 17 of 68 (25.0%) were *STIM1* low-expression.

To examine the clinicopathological role of *STIM1* in CRC, summaries (Table 1) and associations (Table 2) between *STIM1* z-scores and several clinical features were separately examined in COADs and READs. As shown in Table 2, a correlation between *STIM1* and lymphatic invasion was observed (*p* = 0.0253, odds ratio (OR) = 1.4515, 95% confidence interval (CI) = 1.06~2.03) in COADs. *STIM1* also showed a modest borderline significant correlation with the vascular invasion status (OR = 1.4474, 95% CI = 0.99~2.15), but a significant threshold was not reached (*p* = 0.0593). However, there were no statistically significant associations of *STIM1* with the disease stage, tumor stage, lymph node spread status, or distant metastasis status. In contrast, READs did not reach a significant threshold in either lymphatic invasion or vascular invasion, which implied a possible significance of STIM's role in COADs but not ROADs, further suggesting hyperactivation of the SOCE pathway in COAD patients.

**Table 1:** Clinicopathological features of 222 colorectal cancer patients

*Characteristics*	*Total cases N*	*COADs N (%)*	*READs N (%)*	*P-value*
*Gender*				
Male	116	79 (68.10)	37 (31.90)	0.771^a^
Female	106	75 (70.75)	31 (29.25)	
*Age (years)*				
*Mean ± SD*	222	70.66 ± 11.65	66.63 ± 10.76	0.004^b^
Range		36–90	35–89	
*Depth of tumor invasion*				
T1 + T2	55	35 (63.64)	20 (36.36)	0.396^a^
T3 + T4	149	105 (70.47)	44 (29.53)	
*Lymph node metastasis*				
N0	136	94 (69.12)	42 (30.88)	0.839^a^
N1	42	28 (66.67)	14 (33.33)	
N2	44	32 (72.73)	12 (27.27)	
*Distant Metastasis*				
No	185	129 (69.73)	56 (30.27)	0.552^a^
Yes	34	22 (64.71)	12 (35.29)	
*Stage (UICC)*				
I + II (%)	131	91 (69.47)	40 (30.53)	1.000^a^
III + IV (%)	88	61 (69.32)	27 (30.68)	
*Vascular invasion*				
No	153	106 (69.28)	47 (30.72)	0.730^a^
Yes	51	34 (66.67)	17 (33.33)	
*Lymphatic invasion*				
No	101	71 (70.30)	30 (29.70)	0.882^a^
Yes	113	78 (69.03)	35 (30.97)	

Abbrevations: SD, standard deviation; COAD, colon adenocarcinoma; READ, rectal adenocarcinoma; N, number.

a *P*-values were calculated by Fisher's exact test.

b *P*-value was calculated by Wilcoxon test.

**Table 2:** Logistic regression analysis between STIM1 expression value to clinical features in colon adenocarcinoma (COAD) and rectum adenocarcinoma (READ) patients

	COAD^a^					READ^a^			
	No. of pts	OR^b^	95% CI^c^	*P*-value		No. of pts	OR^b^	95% CI^c^	*P*-value
*Stage^d^*	0: 91 1: 61	0.9450	0.69–1.28	0.7176		0: 40 1: 27	0.9642	0.55–1.67	0.8961
*Depth of tumor invasion^e^*	0: 49 1: 105	1.0534	0.76–1.45	0.7499		0: 24 1: 44	1.4404	0.81–2.72	0.2380
*Lymph node metastasis^f^*	N0: 94 N1: 28 N2: 32	N1: 0.8081 N2: 1.2040	N1: 0.54–1.21 N2: 0.82–1.77	N1: 0.2982 N2: 0.3438		N0: 42 N1: 14 N2: 12	N1: 1.1869 N2: 0.6230	N1: 0.62–2.26 N2: 0.28–1.39	N1: 0.6012 N2: 0.2468
*Distant Metastasis^g^*	M0: 129 M1: 22	1.3144	0.86–2.04	0.2138		M0: 56 M1: 12	0.9040	0.42–1.81	0.7834
*Vascular Invasion*	No: 106 Yes: 34	1.4474	0.99–2.15	0.0593		No: 47 Yes: 17	0.7759	0.38–1.53	0.4738
*Lymphatic Invasion*	No: 72 Yes: 77	1.4515	1.06–2.03	0.0253*		No: 30 Yes: 35	0.7167	0.38–1.30	0.2817

a Z-scores were calculated from *STIM1* expression value in 154 COADs and 68 READs patients recruited by TCGA project.

b Odds ratio was calculated with using exp(*β*).

c 95% Confidence intervals.

d COAD and READ patients with Stage I, IIA and IIB were categorized as “0” group versus “1” group that included patients on Stage IIIA, IIIB, IIIC, IV and IVA.

e COAD and READ patients with T1, T2 were categorized as “0” group versus “1” group that included patients on T3, T4a and T4b.

f Multinomial distribution is fitted. N1/N1a/N1b vs N0 and N2/N2a vs N0.

g M0 vs M1/M1a. Significant *P*-value was in bold*.

### STIM1-associated genes differentially expressed in COADs but not READs

COAD and READ patients were further categorized into a *STIM1* overexpression group and *STIM1* low-expression group. Patients with *STIM1* z-scores of >+1 were categorized into the *STIM1* overexpression group (*STIM1*+), and patients with *STIM1* z-scores of <-1 were categorized into the *STIM1* low-expression group (*STIM1*-). According to these criteria, 47 COAD patients (including 27 *STIM1* overexpression and 20 *STIM1* low-expression) and 22 READ patients (including 5 *STIM1* overexpression and 17 *STIM1* low-expression) were subjected to a microarray analysis. DEGs were identified in 69 CRC patients' microarray data. Moderated t-statistics were calculated to identify DEGs in each CRC subtype. In COAD patients, 306 upregulated DEGs and 139 downregulated DEGs were identified and fulfilled the FDR-adjusted *p* value criteria of < 0.1 among the *STIM1* overexpression group and *STIM1* low-expression group (Supplementary Table S1A). Intriguingly, neither significant upregulated nor downregulated DEGs were detected in READ patients. This implied that *STIM1* could be an important marker to distinguish COAD patients from READ patients.

**Figure 2:**
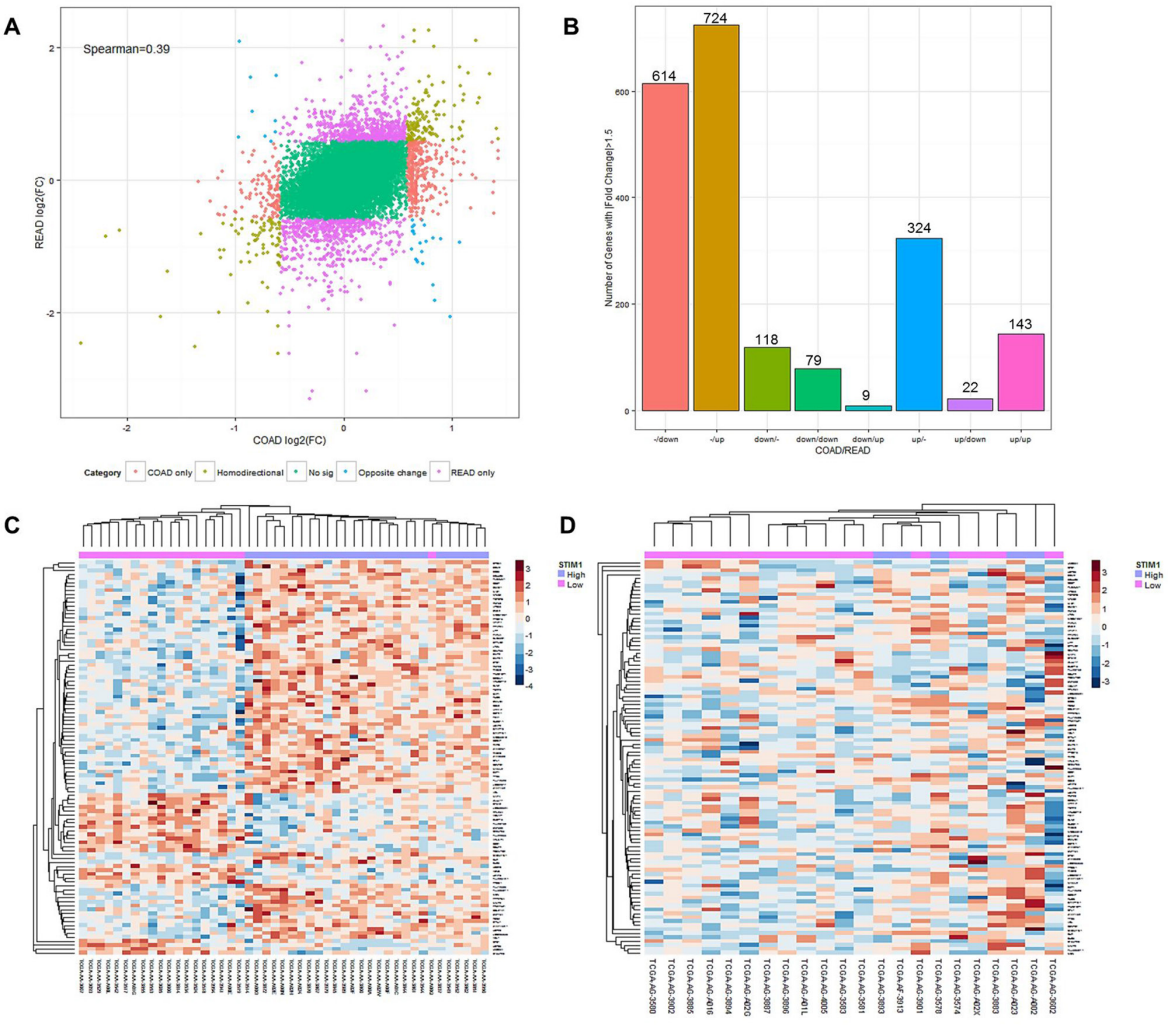
Expression landscape of colorectal cancer subtypes, colon adenocarcinomas (COADs) and rectal adenocarcinomas (READs) **A.** Starburst plot of each gene mapped according to its log2 multiple of change value in COADs (x-axis) and READs (y-axis). Genes with |log2 multiple of change| of >1.5 were selected to examine co-expression patterns. Red: genes up- or downregulated only in COADs. Purple: genes up- or-downregulated only in READs. Brown: genes changing in the same direction in both COADs and READs. Blue: genes changing in opposite directions in COADs and READs. The Spearman rank correlation of coefficient of expression value between COADs and READs was 0.39. **B.** Summarized histogram of selected genes in COADs and READs based on the criterion of a |log2 multiple of change| of >1.5. **C.** Heatmap of the top 100 most variant differentially expressed genes (DEGs) in COADs based on the microarray profile. Each row represents the top 100 most significant DEGs, and each column shows patient samples. A hierarchical clustering analysis was performed, and patient information based on *STIM1* expression status was mapped. **D.** Heatmap of the top 100 most variant DEGs in READs based on the microarray profile. Each row represents the top 100 most significant DEGs, and each column shows patient samples. A hierarchical clustering analysis was performed, and patient information based on the *STIM1* expression status was mapped.

We further identified co-expression patterns between COAD and READ patients. Gene lists between COAD and READ patients were compared using a log2 multiple of change cutoff of ± 1.5 (Figure 2A). Numbers of genes that showed a homodirectional pattern and opposite changes, and were uniquely differentially expressed in COADs or READs were quantified (Figure 2B). In 2033 selected genes based on log2 multiples of change, 614 (30.2%) and 724 (35.6%) were respectively downregulated and upregulated in only READs. In only COADs, 118 (5.8%) and 324 (15.9%) were respectively downregulated and upregulated. There were 79 (3.9%) and 143 (7.0%) genes which respectively showed downregulation and upregulation in both CRC subtypes (homodirectional); while 9 (0.44%) and 22 (1.1%) showed opposite directions in expression patterns across CRC subtypes (Supplementary Table S2). The Spearman rank correlation test revealed a correlation coefficient of 0.39 (*p* < 0.01), indicating a low similarity of expression profiles between COADs and READs. Unsupervised agglomerative hierarchical clustering analyses were conducted to clarify the aggregative effect of *STIM1* expression patterns based on microarray profiles. The top 100 most significantly DEGs identified by the FDR-adjusted *p* value in COADs were clustered, and results are shown for each CRC subtype. As READs showed no significantly up- or downregulated DEGs, no genes were selected for clustering. In COADs, a clear clustering pattern was observed, and a gathering configuration on *STIM1* overexpression patients and *STIM1* low-expression patients was clearly distinguishable (Figure 2C), revealing a highly similar within-group expression pattern in COAD patients. However, a recognizable pattern was no longer visible in READs when we used the same top 100 most significant DEGs for READ clustering (Figure 2D). This result indicated that remarkable latent molecular signatures could be used to distinguish COADs and READs.

Following the clustering analysis, a two-step NMF was carried out against the top 100 DEGs. First, expression values were transformed into non-negative values (Supplementary Figure S1A). Then, Brunet et al.′s algorithm [8] was performed on the factorization rank between 2 and 6 in 200 runs to identify the optimal number of clusters. With the aid of visualization by a consensus matrix, the strongest consensus signature was observed with a factorization rank of 2 (Supplementary Figure S1B). The result indicated that the NMF algorithm could attain good clustering stability in a factorization rank of 2 that consistently clustered patients in each run based on *STIM1* expression features. This was consistent with the hierarchical clustering result, that COAD patients were well-categorized into *STIM1* high-expression and *STIM1* low-expression groups based on two metagenes (Supplementary Figure S1C).

A series of analyses revealed that (i) the underlying molecular schemes of COADs and READs differed; (ii) *STIM1* could be a biomarker for COADs but not READs, and (iii) there were differences in fundamental biological pathways between COADs and READs.

### Prospecting underlying biological features in COADs and READs

For microarray expression profiles of COAD and READ patients, all signatures were included in the GSEA to detect coordinated changes in the same BP terms. Outcome enrichment scores were further normalized for comparisons (Supplementary Tables S3, S4). After filtering, differences in normalized enrichment scores were compared between COADs and READs (Supplementary Figure S2). Note that only the BP terms with *a* value of NES_COAD_-NES_READ_ of > 0 (which means a positive value) were of interest. Therefore, BP terms identified to be significantly enriched in COADs compared to READs were “immune system process” (GO:0002376), “regulation of cell migration” (GO:0030334) and “regulation of cell motility” (GO:2000145) (Supplementary Figure S3). In particular, the BP terms of “regulation of cell migration” and “regulation of cell motility” could work in concert with clinical features of *STIM1*, which is associated with lymphatic invasion in COAD patients. Specifically, the enriched BP terms provided support for the involvement of *STIM1* and *STIM1*-related molecular signatures in tumor invasion progress. This result also indicated a similar enriched BP (which represented common CRC or cancer features) but different subtype-related molecular signatures (which represented subtype-enriched BPs) in CRC patients.

### Validation of distinctive STIM1 roles in CRC subtypes by RNA-sequencing analysis and a pathway topology-based approach

**Figure 3:**
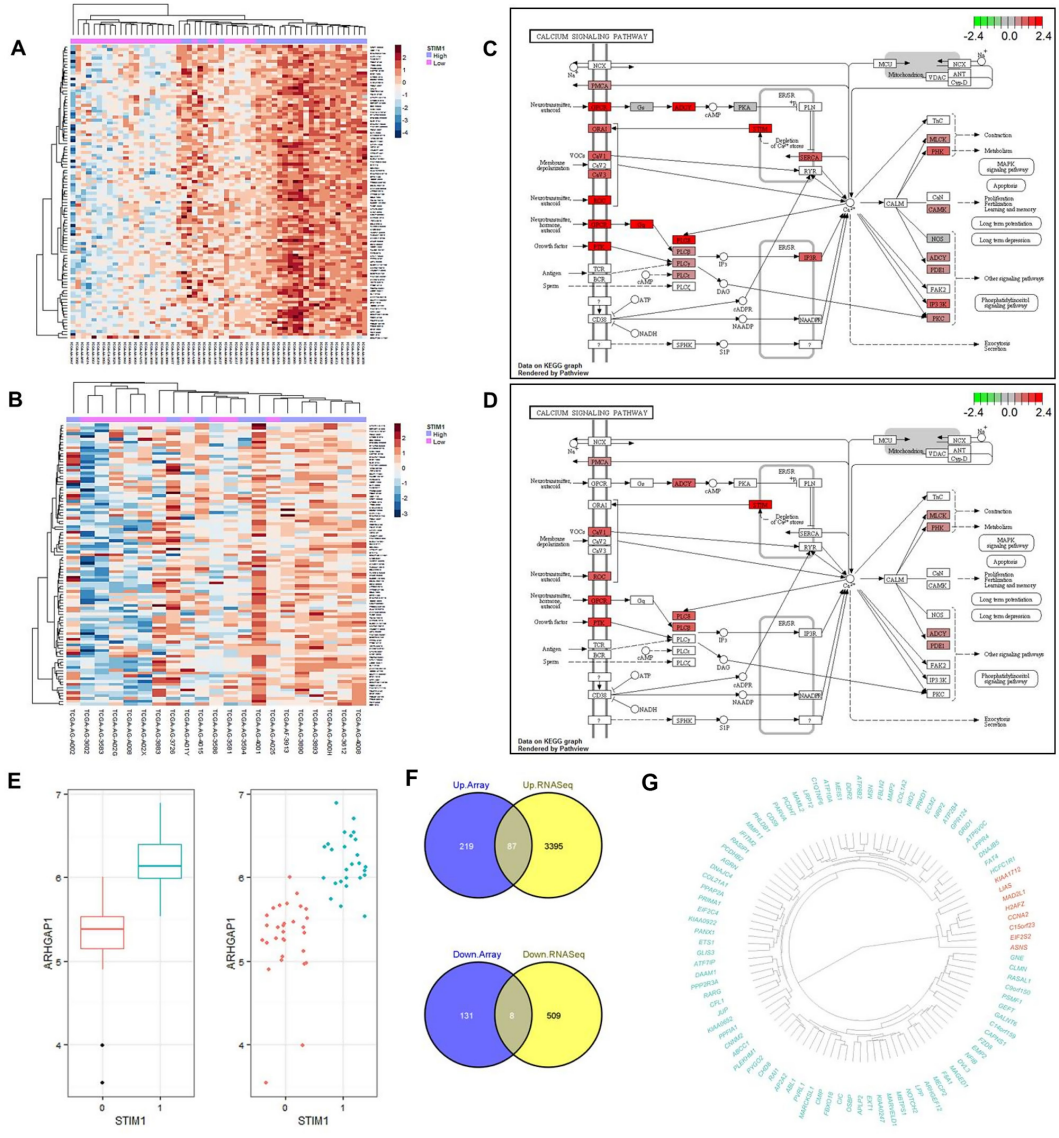
Validation of differential expression results by RNA-sequencing platform and a signaling pathway impact analysis (SPIA) **A.** Heatmap of the top 100 most variant differentially expressed genes (DEGs) in colon adenocarcinomas (COADs) based on the RNA sequencing profile. Each row represents the top 100 most significant DEGs, and each column shows patient samples. A hierarchical clustering analysis was performed, and patient information based on the *STIM1* expression status was mapped. **B.** Heatmap of the top 100 most variant DEGs in rectal adenocarcinomas (READs) based on the RNA sequencing profile. Each row represents the top 100 most significant DEGs, and each column shows patient samples. A hierarchical clustering analysis was performed, and patient information based on the *STIM1* expression status was mapped. **C.** Calcium signaling pathway of COADs based on the KEGG graph. The degrees of log2 multiples of change were mapped by red or green color based on the direction of the log2 multiple of change. **D.** Calcium signaling pathway of READs based on the KEGG graph. The degrees of log2 multiples of change were mapped by red or green color based on the direction of the log2 multiple of change. **E.** Whisker boxplot and jitter plot summarizing differences of normalized *ARHGAP1* expression levels in *STIM1* overexpression and *STIM1* low-expression groups (false discovery rate-adjusted *p* = 8.55 × 10^−7^). In x-axis, 1: *STIM1* overexpression; 0: *STIM1* under-expression. **F.** Venn diagrams showing the number of intersecting significantly differentially up- or downregulated genes across the microarray platform and RNA sequencing platform in COADs. Plots were constructed using Venny (http://bioinfogp.cnb.csic.es/tools/venny/index.html). **G.** Dendrogram of 95 validated genes (green: 87 upregulated genes; red: 8 downregulated genes) constructed by a dissimilarity matrix of expression values based on Pearson's correlations. Genes were ordered by a hierarchical clustering based on the Euclidean distance and average linkage.

RNA-sequence RPKM values were analyzed to elucidate the subtype-specific effect of *STIM1* in CRC. In total, 56 COAD patients (including 26 *STIM1* overexpression and 30 *STIM1* low-expression) and 21 READ patients (including 11 *STIM1* overexpression and 10 *STIM1* low-expression) were investigated. To harmonize the RPKM value to downstream differential expression identification, we rounded RPKM values below 0.1 to prevent the deviation caused by low-coverage genes and then used a log2 transformation to fit the rounded RPKM values into a normal distribution. After normalization, a moderated *t*-test was applied to identify DEGs and an FDR-adjusted *p* value threshold of 0.1 was applied for filtering. Surprisingly, the RNA-sequencing profile identified 3482 upregulated DEGs in COADs but 0 in READs, and 517 downregulated DEGs in COADs and 0 in READs (Supplementary Table S1B), which is similar in character to the microarray profile. A corresponding hierarchical clustering analysis also disclosed a discernible pattern in COADs (Figure 3A) but not READs (Figure 3B).

The SPIA [9], based on the KEGG pathway database [10], was applied to gain biological insights into calcium signaling pathways in COADs and READs. In COADs, the calcium signaling pathway was significantly overrepresented (Figure 3C, Supplementary Table S5A, Supplementary Figure S4A), and the corresponding FDR-adjusted *p* value was equal to 0.0104 (Supplementary Table S6). Note that the aberration of the calcium signaling pathway in COADs was supported by over-representation evidence but not perturbation evidence. Comparatively, the calcium signaling pathway met neither the over-representation criterion nor the perturbation criterion (Figure 3D, Supplementary Table S5B, Supplementary Figure S4B) in READs.

Furthermore, in COADs, *STIM1* overexpression was most highly correlated with upregulation of *ARHGAP1*/*Cdc42GAP* (FDR-adjusted *p* = 8.55 × 10^−7^, estimated beta = 14.64), a Rho GTPase-activating protein which participates in RhoGTPase signaling pathway that is implicated in the EMT, showed extensive function on regulating cell proliferation, migration, invasion, adhesion, and apoptosis [11]. Although the role of *ARHGAP1* in CRC has remained obscure, alteration of *ARHGAP1* might contribute to tumor aggression (Figure 3E).

Concordant expressions between different platforms (microarray and RNA-sequencing) were analyzed. We intersected upregulated DEGs and downregulated DEGs across different platforms and found 87 consistently upregulated and 8 consistently downregulated DEGs (Figure 3F). In other words, these 95 DEGs were successfully validated across different expression platforms, and the corresponding genes are shown in Figure 3G.

These results indicated a significant enrichment of the calcium signaling pathway in COADs but not ROADs, further supporting the inference of CRC subtype-specific characteristics of *STIM1*-mediated SOCE pathway changes, and the relevance of *STIM1*-associated Ca^2+^ signaling to COADs but not READs.

### Association of STIM1 overexpression with miRNA in COAD and READ patients

Besides mRNA expression levels, miRNA profiles of the CRC cohort were also studied. COAD and READ patients with available miRNA sequencing data were stratified into a *STIM1* overexpression group and *STIM1* low-expression group. In total, 80 COAD patients (including 40 *STIM1* overexpression and 40 *STIM1* low-expression) and 32 READ patients (including 11 *STIM1* overexpression and 21 *STIM1* low-expression) were selected.

**Figure 4:**
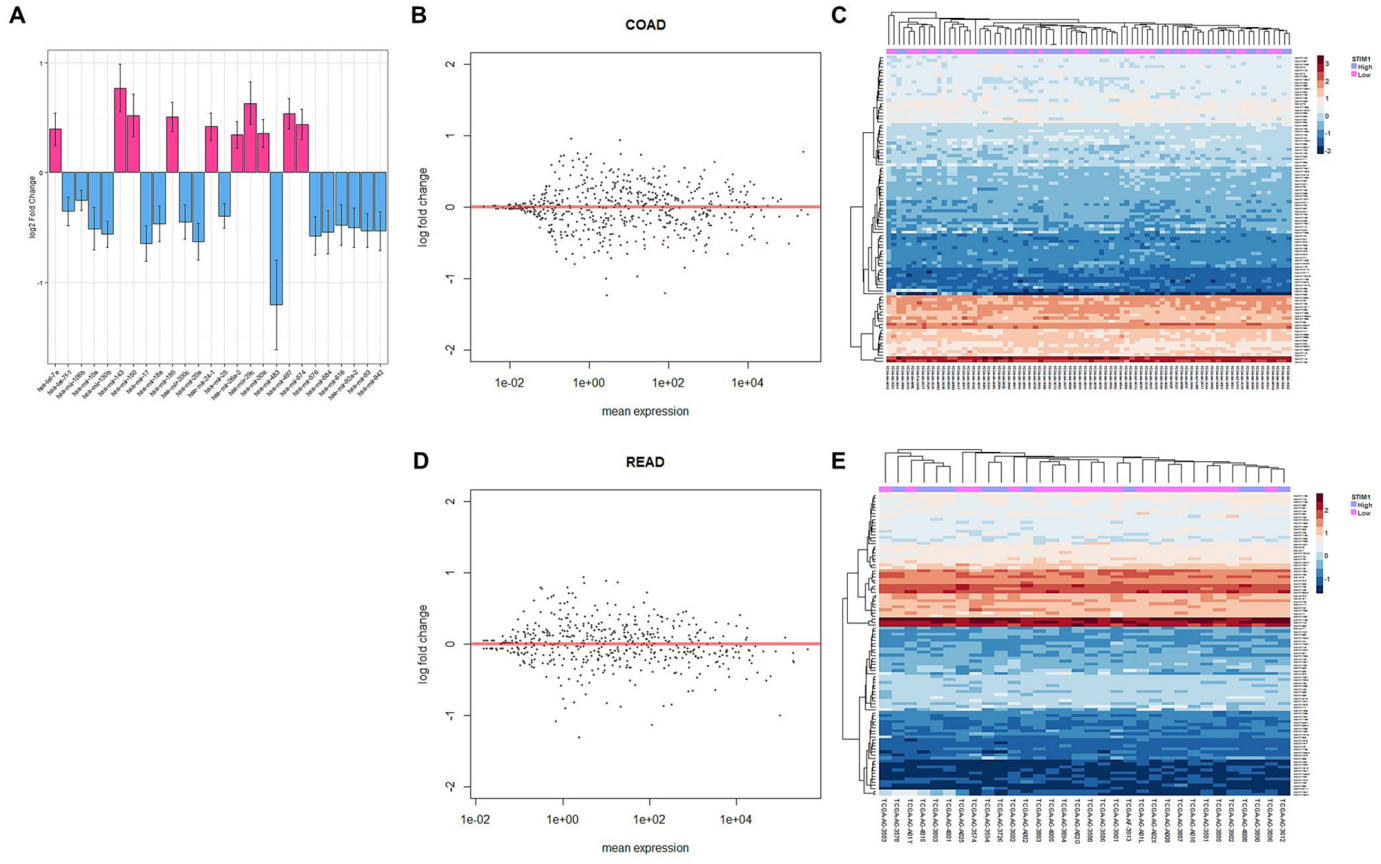
Analysis of colorectal cancer (CRC) patients' micro (mi)RNA expression profiles **A.** Bar plot of significantly expressed miRNAs in colonic adenocarcinoma (COAD) patients and the corresponding log2 multiple of change value. The estimated standard error is represented by bars. **B.** MA plot of all miRNAs in COAD patients. The multiple of change was calculated based on the contrast between *STIM1* overexpression and *STIM1* low-expression, and miRNAs that passed the false discovery rate (FDR) threshold (FDR < 0.1) are in red color. **C.** Heatmap of the top 100 most variant miRNAs in COADs. Each row represents the top 100 most significant miRNAs, and each column shows patient samples. A hierarchical clustering analysis was performed, and patient information based on the *STIM1* expression status was mapped. **D.** MA plot of all miRNAs in rectal adenocarcinoma (READ) patients. The multiple of change was calculated based on the contrast between *STIM1* overexpression and *STIM1* low-expression, and miRNAs that passed the FDR threshold (FDR < 0.1) are in red color. **E.** Heatmap of the top 100 most variant miRNAs in READs. Each row represents the top 100 most significant miRNAs, and each column shows patient samples. A hierarchical clustering analysis was performed, and patient information based on the *STIM1* expression status was mapped.

The contrast based on the presence or absence of *STIM1* overexpression was applied to calculate multiples of change of miRNA, and a negative binomial distribution was fitted to account for the non-normality and dependency of the variance on the mean [12]. Having estimated the multiples of change and calculated corresponding *p* values of miRNA in each CRC subtype, a FDR-adjusted *p* value threshold of 0.1 was applied to identify differentially expressed miRNAs across COADs and READs. As a result, 10 upregulated and 16 downregulated miRNAs were identified in COAD patients (Figure 4A, Supplementary Table S7A); while only 2 downregulated miRNAs (hsa-miR-1978 and hsa-miR-203) showed a significant correlation in READ patients (Supplementary Table S7B).

The following regularized log transformation and hierarchical clustering analysis were carried out to inspect comprehensive miRNA regulation of COADs (Figure 4B–4C) and READs (Figure 4D–4E). However, no distinctive pattern was observed in COADs and READs, indicating a modest regulatory role of *STIM1* overexpression in the overall miRNA profile. In spite of the fact of undetectable global interference of miRNA expression, *STIM1* overexpression indeed affected a small number of miRNAs in COADs and READs (Supplementary Table S7C).

### Identification of prognostic mRNA/miRNA signatures in COADs for clinical outcomes across different clinical subclasses

**Figure 5:**
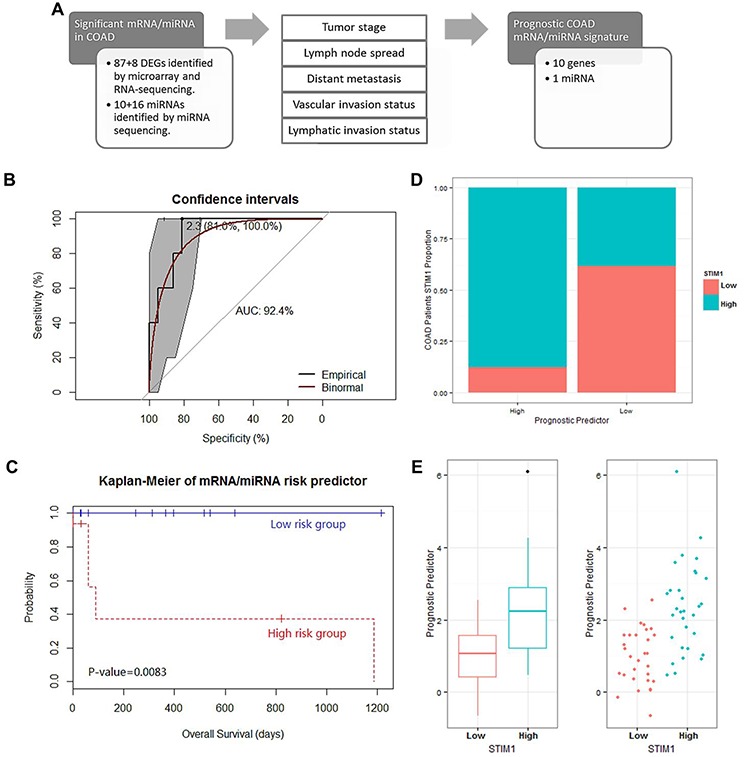
Analysis of the prognostic mRNA/micro (mi)RNA signature identification in 63 colon adenocarcinoma (COAD) patients and their performance in survival prediction **A.** Scheme of the survival analysis strategy. A survival analysis was performed on the following clinical subclasses: tumor stage, lymph node pathologic spread, distant metastasis, vascular invasion, and lymphatic invasion. In total, 95 mRNAs and 26 miRNAs that were significant in at least 2 clinical subclasses were considered significantly associated with patient survival. **B.** Receiver operating characteristic (ROC) curve of prognostic predictors with 1-specificity as the x-axis and sensitivity as the y-axis. The 95% confidence interval of the ROC curve was bootstrapped by DeLong's method to confirm the significance of the ROC curve. The best threshold (prognostic predictor = 2.3) and corresponding specificity and sensitivity (81.0% and 100.0%, respectively) are shown. In addition, the area under the ROC (AUROC) curve of 92.4% is shown. The binomial smoothed line is shown in red by the binomial method. **C.** Kaplan-Meier curve for overall survival for dichotomized prognostic predictor. *p* values were calculated by the Cox regression analysis of *STIM1* overexpression group and *STIM1* low-expression group. **D.** Correlation between the presence or absence of *STIM1* overexpression and the dichotomized prognostic predictor. **E.** Whisker boxplot and jitter plot for comparison between the presence or absence of *STIM1* overexpression and the continuous prognostic predictor.

Having determined the 95 genes that were consistently detected by microarray and RNA-sequencing platforms and 16 miRNAs detected by miRNA sequencing in COADs, we conducted a stringent multistep clinical subclass-based survival analysis, which was similar to the strategy proposed by Volinia et al. [13], to identify COAD prognostic mRNA/miRNA signatures (Figure 5A). Thus, survival analyses were separately adjusted according to clinical profiles (T stage, N stage, M stage, and vascular- and lymphatic-invasion status). Furthermore, only mRNA or miRNA that showed *p* values of < 0.05 in at least two clinical subclasses were considered significant. Therefore, mRNA or miRNA identified to be significant indicated that these signatures were correlated with the patient survival rate after adjusting for several clinical subclasses.

Consequently, 10 transcripts, including *PANX1*, *ETS1*, *EXT1*, *C1QTNF6*, *PCDH7*, *GLIS3*, *MARVELD1*, *PPP2R3A*, *KIAA0247*, and *LRP12*, and 1 miRNA (*hsa-miR-195*, Supplementary Figure S5) successfully passed the multistep procedure and were related to clinical outcomes across COAD patients (Supplementary Table S8).

In addition, we constructed a PCA algorithm to transform these 11 prognostic signatures into a linear form (a so-called prognostic predictor, Supplementary Figure S6A). The model performance was assessed by the AUROC curve. The ROC curve was used to define the best threshold, and then patients were stratified into high-risk or low-risk groups based on this threshold. In COAD patients, the prognostic predictor value of each patient was calculated by the model based on the expression value, and the best threshold (prognostic predictor = 2.3) was calculated by the ROC curve (with a sensitivity of 100.0% and a specificity of 81.0%). In addition, the AUROC curve of 92.4% revealed good performance on survival prediction using the prognostic predictor (Figure 5B). Thus, the survival rates of COAD patients (consisting of *STIM1* overexpression and *STIM1* low-expression) categorized into two groups based on the prognostic predictor were compared. The Kaplan-Meier estimator revealed a significant difference in survival rates across the 2 groups (Cox-proportional hazard *p* = 0.0083, Figure 5C). Moreover, we collected all available COAD patients without considering the *STIM1* expression value to carry out a prognostic predictor-based survival analysis, and results revealed a decline in the ROC performance (AUROC = 65.8%, Supplementary Figure S6B). The corresponding Kaplan-Meier estimator also revealed a decrease in the ability to separate high-risk and low-risk groups, although the prognostic predictor still attained a significant effect in survival prediction (Cox-proportional hazard *p* = 0.022, Supplementary Figure S6C). These results indicated that the *STIM1*-related mRNA/miRNA signature participated in the transcriptomic foundation for survival, but could only explain a portion of the overall survival in all COAD patients.

Finally, to elaborate the association between the prognostic predictor and *STIM1* expression status, we compared prognostic predictor values between the *STIM1* overexpression and *STIM1* low-expression groups. COAD patients who fell within the high-risk group (prognostic predictor > 2.3) consisted of a large portion of the *STIM1* overexpression group (87.5%, 14 of 16), whereas the low-risk group consisted of a larger number of the *STIM1* low-expression group (61.7%, 29 of 47) (approximate Pearson's Chi-squared *p* = 0.001, Figure 5D). An in-depth analysis also revealed a strong difference in prognostic predictor value between *STIM1* expression groups (exact Wilcoxon Mann-Whitney rank sum *p* = 1.39 × 10^−5^, Figure 5E). Therefore, *STIM1* overexpression and *STIM1*-related molecular signatures were tightly correlated with COAD patient prognoses, and this phenomenon was enormously significant especially in the *STIM1* overexpression group versus the *STIM1* low-expression group.

## DISCUSSION

Our findings have several important implications for understanding *STIM1* overexpression in CRC. First, we clearly determined distinct transcriptomic subtypes of CRC and documented the existence of a subtype-specific *STIM1* role in CRC. An aberration of the SOCE pathway was previously described in CRC, and the *STIM1*-mediated Ca^2+^ oscillation was linked to tumor aggressiveness. However, the existence of CRC-subtype specific characteristics of the *STIM1*-mediated SOCE pathway remained elusive before this study. The combination of a dual-platform (microarray and RNA sequencing) genome-wide approach robustly discriminated *STIM1*-associated signatures as a feature of COADs but not READs in CRC patients. COAD patients have a profound increase in morbidity compared to READ patients, but the underlying global molecular mechanisms corresponding to this difference are unclear.

In this study, we observed a correlation between *STIM1* expression values (z score) and lymphatic invasion in COADs. However, no significant correlation between *STIM1* level and TNM and disease stage was identified. In compared with Wang et al. works [14], the *STIM1* staining intensity was correlated with metastasis and disease stage. We noted that different *STIM1* measurement approaches (mRNA transcript level by microarray versus protein staining) may contribute to the inconsistent findings.

In cancer cells, secondary messenger Ca^2+^ regulation of widespread physiological and pathological processes include tumor dissemination. The SOCE pathway was identified as the major Ca^2+^ entry mechanism in tumor cells. A decrease in the ER Ca^2+^ concentration further triggers *STIM1*, a calcium sensor, to aggregate and translocate to cell membranes. Orai1, a plasma membrane store-operated calcium channel, is activated by *STIM1* to allow Ca^2+^ influx. Consequently, *STIM1*-regulated Ca^2+^ influx facilitates focal adhesion turnover [5] and controls invadopodium formation and activity [4]. Significantly, *STIM1* regulates cancer cell migration and invasion ability, and this concept was supported by our data, which showed a correlation between elevated *STIM1* expression levels and an enhanced lymphatic invasion status in COAD patients. Using computational tools, we also observed a significant SOCE pathway perturbation resulting from *STIM1* overexpression in COADs, which modulates the biological processes of cell migration and cell motility. Therefore, *STIM1* overexpression implicates a hyperactive SOCE pathway in COADs and demonstrates a higher propensity for invasion. However, comparing between *STIM1* overexpression and *STIM1* low-expression groups of READ patients did not reveal dysregulation of the SOCE pathway, unraveling a minor role of *STIM1* and the SOCE pathway in ROAD tumor dissemination. In Li et al.'s study [3], the calcium signaling pathway was one of the downregulated pathways in READs, which was consistent with our SPIA results: a negative direction (inhibition) of the SOCE pathway perturbation. Comparing differences in *STIM1* expression values in CRC subtypes indicated a slight increase in *STIM1* overexpression patients in COADs, and a slight increase in *STIM1* low-expression patients in READs. These results should be carefully interpreted, as the divergence of the *STIM1* overexpression status in CRC subtypes was small, and the difference in the degree of impact of the SOCE pathway in CRC subtypes might not be simply and completely attributed to discrepancies in *STIM1* distribution. A possible explanation of the dissimilarity expression profiles across COADs and READs could be that *STIM1* overexpression might be due to a consequence of an aberration of different multiple upstream signaling pathways, and downstream influences of *STIM1*-mediated Ca^2+^ changes were multifaceted and multifactorial.

In the GSEA analysis, the ectopic *STIM1* profile showed positive enrichment in immune system processes in COADs and a negative correlation was noted in READs. In pathophysiological aspects, *STIM1*-mediated Ca^2+^ signaling and the SOCE pathway play critical roles in regulating immune responses, and activation of the SOCE pathway through *STIM1* overexpression dramatically worsens the proinflammatory status [15, 16]. Increasing evidence indicates that *STIM1*-mediated cyclooxygenase (COX)-2 overexpression, an important inducible proinflammatory enzyme, might exacerbate tumor migration and progression [17]. In addition, impairment of regulation of the immune system is tightly bound to clinical outcomes of CRC. Therefore, our results suggest that *STIM1*-mediated Ca^2+^ signaling and *STIM1* overexpression might be prospective therapeutic targets for COAD treatment. In the network analysis, the module included genes correlated with lymphatic invasion that were also identified. Significantly, a large number of proteins in this network (*MAPK9*, *MAP2K4*, *MAPK10*, *MAP4*, *MARK4*, *TGFBR2*, and so on) were associated with mitogen-activated protein kinase (MAPK) pathways, which are well-known for their role in CRC. However, associations between *STIM1* and MAPK signaling pathways are ill-defined. Our data illustrated a possibility of interaction between the calcium signaling pathway and MAPK signaling pathway in COADs. Furthermore, Fan et al. showed the migration-promoting effect of a CRC cell line of *SERCA* overexpression via activation of the MAPK signaling pathway, further suggesting direct crosstalk between Ca^2+^ signaling and MAPK signaling [18]. In addition, alpha-actinin 1 (*ACTN1*), one of the genes identified in the network, is associated with focal adhesion formation, and its phosphorylation modulates pressure-induced adhesion in colon cancer cells [19]. Activating transcription factor 7 (*ATF7IP*), another transcription factor proven to be related to lymphatic invasion in our study, is involved in telomerase expression mediated by Sp1 [20]. In short, we successfully identified an integrated network module which was mostly positively correlated with a dysregulated *STIM1* signature, which constituted the *STIM1*-associated invasiveness nature of COADs.

Beyond the mRNA data, COAD-associated miRNAs were discovered by a miRNA sequencing analysis. Specifically, loss of *hsa-miR-10a* that targets *KLF4*, as seen in *STIM1* overexpression patients, led to upregulation of LPO and initiation of colorectal carcinomas [21]. *hsa-miR-130b* was reported to suppress CRC invasion and migration by downregulating integrin β1 [22], and downregulation of *hsa-miR-130b* was observed in the *STIM1* overexpression group in COADs. *hsa-miR-18a*, that targets CDC42 and acts as a tumor suppressor, was significantly downregulated in *STIM1*-enriched patients [23]. *hsa-miR-200c*, which was downregulated in the *STIM1* overexpression group, is associated with proliferation, migration, and invasion in CRC cell lines [24]. In addition, upregulation of *ANGPTL2*, which is associated with downregulation of *hsa-miR-25*, was correlated with reductions in the invasive and migratory abilities of human CRC [25]. Furthermore, downregulation of *hsa-miR-93* was observed, which promotes colon cancer development via upregulation of the Wnt/β-catenin pathway [26]. In READs, *hsa-miR-203* downregulation is associated with upregulation of Snail and improved invasion or metastasis potential of CRC cell lines [27].

In this study, we made our hypothesis based on cancer cells but not stroma cells in cancer tissues. The limitation of our study is that we could not clarify the STIM1 expression in cancer cells or that in stroma cells. However, STIM1 level is unlikely to be dominant in stroma cells because STIM1 was also highly expressed in cancer cells (data not shown).

The genomic basis of prognostic *STIM1*-associated signatures is unclear. Our results demonstrated that dysregulation in the *STIM1*-associated transcriptome explains a proportion of the differences between COADs and READs that underlie these signatures. The compact correlation of these changes with a *STIM1*-concerted expression phenotype plays a critical *STIM1*-centered role in predicting clinical outcomes. In addition, the transcriptome profiles we derived and the associated *STIM1*-associated signatures provide a previously unknown mechanistic link between CRC subtypes with differing invasive behaviors and transcriptional signatures that predict clinical prognostic outcomes. The data herein indicate a straightforward correlation between *STIM1*-mediated Ca^2+^ signaling and wide-ranging signatures known to be associated with tumor aggression, and this implication was only present in COADs. In summary, this study provides a comprehensive transcriptomic framework for understanding *STIM1*-related mRNA/miRNA signatures present in CRC subtypes with differing invasive behaviors, and suggests that Ca^2+^ signaling-targeted therapy may help further perfect the clinical capability to implement precision medicine for CRC patients.

## MATERIALS AND METHODS

### Clinical and integrated profiles of the TCGA CRC cohort and related patients

Having determined patient categorization, we queried COAD and READ samples based on the *STIM1* expression value (z-score) using the cBio Cancer Genomics Portal (http://www.cbioportal.org/). Sample data of different technical platforms (microarray, RNA-sequencing, and miRNA sequencing) were downloaded from the TCGA website (https://tcga-data.nci.nih.gov/tcga/) in data level 3 or a data matrix [7]. Following the procedures of *Tell et al*. [28], transcriptomic profiles were studied in patients of each CRC subtype, and patient categorization was carried out based on *STIM1* z-scores. In this study, we defined CRC patients with a *STIM1* z-score of >+1 as the overexpression group (*STIM1*+), and those with a *STIM1* z-score of <-1 as the low-expression group (*STIM1*-). Simultaneously, comparisons between *STIM1*+ and *STIM1*- were performed across different experimental types.

### Analysis of clinical features of CRC patients

Extended clinical demographics including cancer stage and survival data were acquired from the TCGA data portal. In total, 154 COAD and 68 READ patients were included to assess the correlation between *STIM1* expression values and clinical features. A logistic regression model under a quasibinomial distribution was fitted for the association test in COAD and READ patients. According to the disease stage, patients were categorized into 2 groups (stages I, IIA, and IIB vs. stages IIIA, IIIB, IIIC, IV, and IVA). Similarly, patients were categorized into a T1 and T2 group and a T3, T4a, and T4b group according to the T stage. In addition, a multinomial logistic regression was applied to identify correlations between *STIM1* and the N stage (N0 vs. N1 vs. N2).

### Analysis of microarray and RNA-sequencing profiles of CRC patients

Microarray data including 47 COAD and 22 READ samples and RNA-sequencing data including 56 COAD and 21 READ samples with available *STIM1* expression value were subjected to data sanitization. Then, we imputed missing values by the kth-nearest neighbors (k-NN) algorithm. As the downloaded microarray data had already been normalized, we identified differentially expressed genes (DEGs) using a moderated *t*-test [29] by comparing *STIM1*+ and *STIM1*- patients who had COADs and READs. Significant DEGs were defined as genes having a false discovery rate (FDR)-adjusted *p* value threshold of < 0.1. Unsupervised hierarchical clustering was carried out on COADs and READs with the top 100 most variant DEGs identified in COADs. Note that the top 100 DEGs in READs were not analyzed because none of them passed the statistical cutoff point, and thus the analysis would have been nonsensical. We then applied the non-negative matrix factorization (NMF) method to validate the clustering effect [30] of the top 100 most variant DEGs using Brunet et al.′s algorithm in COADs [8]. In the NMF analysis, 200 runs were iteratively performed to identify the stability of the consensus matrix under different factorization ranks of 2 to 6. For RNA-sequencing data, the value of reads per kilobase per million reads (RPKM) was provided. In order to fit the RPKM to the downstream DEG identification analysis, we normalized the RPKM value with a rounding cutoff of 0.1 and log2 transformation [31]. DEGs were identified by a moderated *t*-test with an FDR-adjusted *p* value threshold of 0.1.

### Gene set enrichment and interactome analysis of DEGs in CRC patients

Following expression analysis, a gene ontology (GO) biological process (BP) term analysis was performed [32]. We separately applied the gene set enrichment and analysis (GSEA) algorithm [33] to identify enriched BP terms in COADs and READs. After permutation, BP terms were filtered by the following criteria in both CRC subtypes: an FDR value of < 5 × 10^−5^, a gene set size of > 150, and an absolute value of subtracted normalized enrichment score (NES) of > 1.2. Remaining BP terms were then compared between COADs and READs. In interactome aspects, a network analysis was conducted to identify differentially expressed network modules corresponding to the lymphatic invasion phenotype (see Supplementary Note) [34]. We used Human Protein Reference Database (HPRD) to provide protein-protein interaction information [35]. We then aggregated *p* values and fit a beta-uniform mixture (BUM) distribution. Scored nodes in the network were then used to find a maximum scoring subnetwork with a heuristic algorithm [36].

### Signaling pathway impact analysis (SPIA) of DEGs in CRC patients

We carried out an signaling pathway impact analysis (SPIA) to analyze the difference of aberrant pathways between COADs and READs using RNA sequencing data [9]. In COADs, 3648 DEGs based on an FDR threshold of 0.1 were selected for the over-representation analysis (ORA), a part of the analytic workflow implemented in SPIA; however, 1215 genes with *p* values of < 0.05 in READs were subjected to an ORA analysis as no gene passed FDR filtering. In addition, the number of bootstrap iterations used to compute the perturbation *p* value was 20,000, and Fisher's combined method was used to combine the over-representation *p* values and perturbation *p* values [37].

### Analysis of miRNA sequencing profile in CRC patients

We tested for associations of miRNA-*STIM1* expression levels by assuming a negative binomial distribution using a generalized linear model [12]. In total, 80 COAD and 32 READ patients were included in the miRNA analysis. The raw miRNA read counts were directly applied for differentially expressed miRNA identification. We set the significance threshold of FDR-adjusted *p* values to 0.1. We then regularized log transformation of count data to undergo downstream heatmap and hierarchical clustering analyses.

### Survival analysis of significant mRNA/miRNA signatures

In COADs, the correlation between RNA expression and overall survival was conducted by a modified strategy which was similar to that proposed by Volinia and Croce [13]. To avoid bias caused by an imputed expression level, we removed patients with at least one missing value of mRNA/miRNA signatures. Hazard ratios were calculated by Cox-proportional hazard coefficients. Clinical covariates were incorporated into the multivariate Cox-proportional hazard model to identify independent molecular RNA signatures. Therefore, the association results were reported as five clinical subclasses: tumor stage, lymph node spread status, distant metastasis, vascular invasion status, and lymphatic invasion status. To evaluate the prognostic value of the identified mRNA/miRNA signatures, we used a principal component analysis (PCA) algorithm to calculate the linear combination of corresponding molecular signatures. The best threshold and performance of prognostic predictor were determined by a receiver operating characteristic (ROC) curve and area under the ROC (AUROC) curve, respectively [38].

### Statistical analysis and annotation

We used R (http://www.r-project.org/; http://cran.r-project.org/) and Bioconductor (http://www.bioconductor.org/) for all analytic workflows in this study.

## SUPPLEMENTARY FIGURES AND TABLES




