# Supplementary material for: Correlation of clinical features and genetic profiles of stromal interaction molecule 1 (STIM1) in colorectal cancers
Source: Oncotarget. 2015 Nov 3;6(39):42169–82. doi: 10.18632/oncotarget.5888 (PMC4747217; doi:10.18632/oncotarget.5888)
Supplement: Supplementary file 1 [file oncotarget-06-42169-s001.pdf]

SUPPLEMENTARY FIGURES AND TABLES

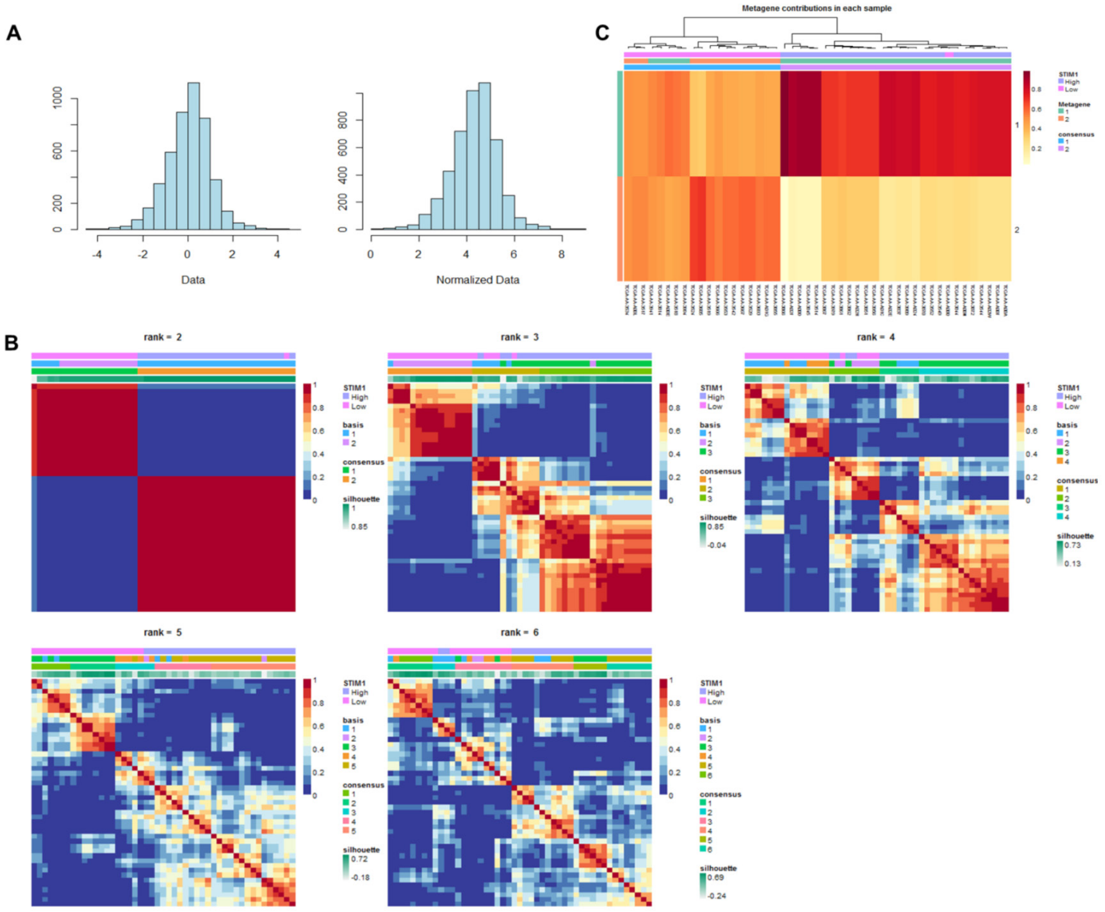

Supplementary Figure S1: Nonnegative matrix factorization results of top 100 most variant DEGs in COAD patients. A. Histograms showed before and after nonnegative data transform of expression value (x-axis). Before transformation, expressions value were ranged between −4 to 4; however, after nonnegative transformation, all expression values are larger than 0. The similar distribution shape indicated the attainment of consistent data structure before and after transformation. **B.** At each factorization rank (2–6), a consensus matrix was plotted by averaging 200 connectivity matrices. The strong consensus for rank number equal to two was indicating a repeatable partitioning of samples into two classes. **C.** Heatmap of metagene matrix was obtained from factorization rank of 2. Each row represented a metagene and each column represented a patient. Patients were ordered by hierarchical clustering based on the Euclidean distance and average linkage.

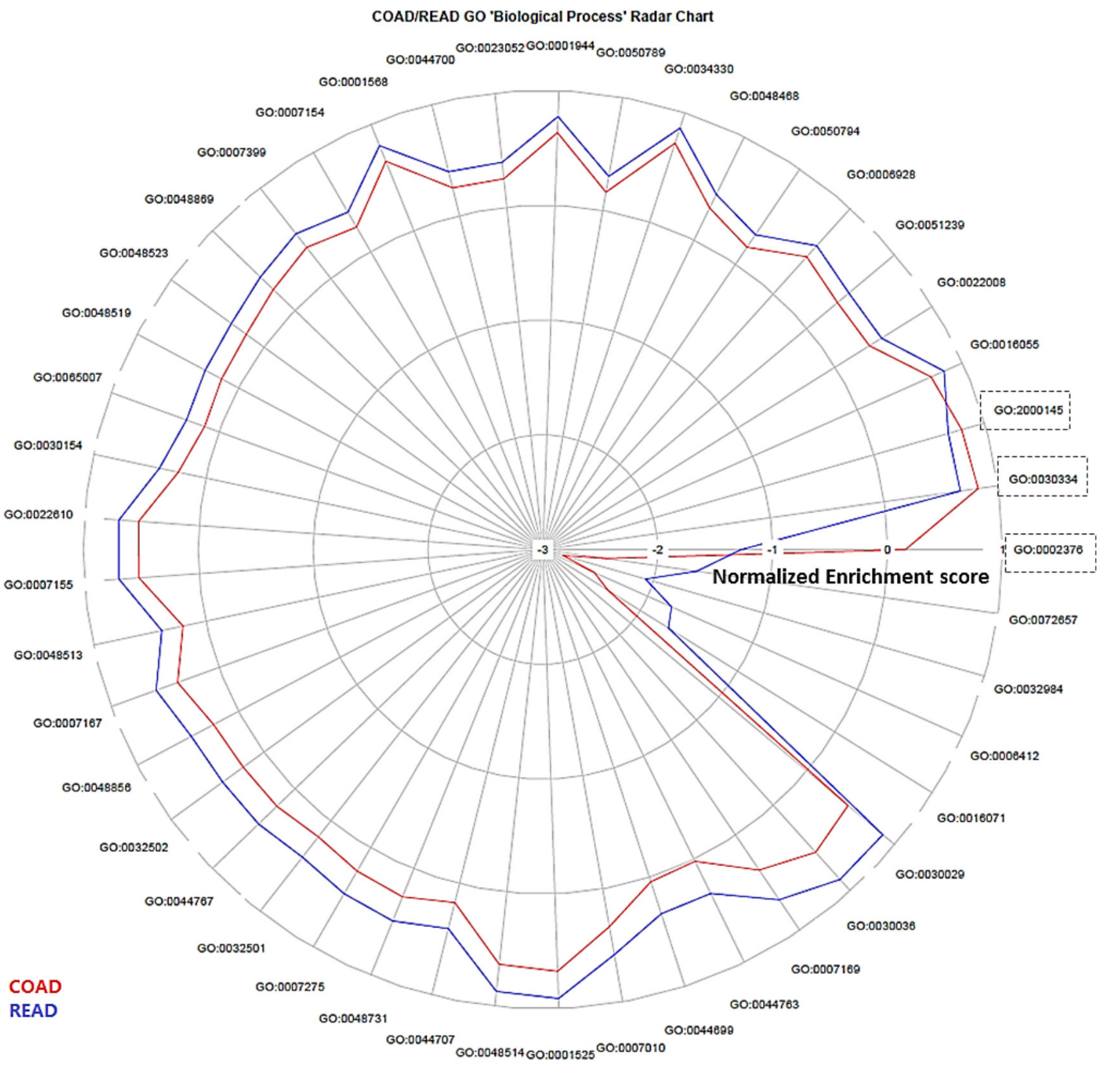

Supplementary Figure S2: Radar chart showed the normalized enrichment score of COADs (red) and READs (blue) in GO terms that passed the described criteria. The GO terms that more overrepresented in COADs than READs were highlighted in black bracket.

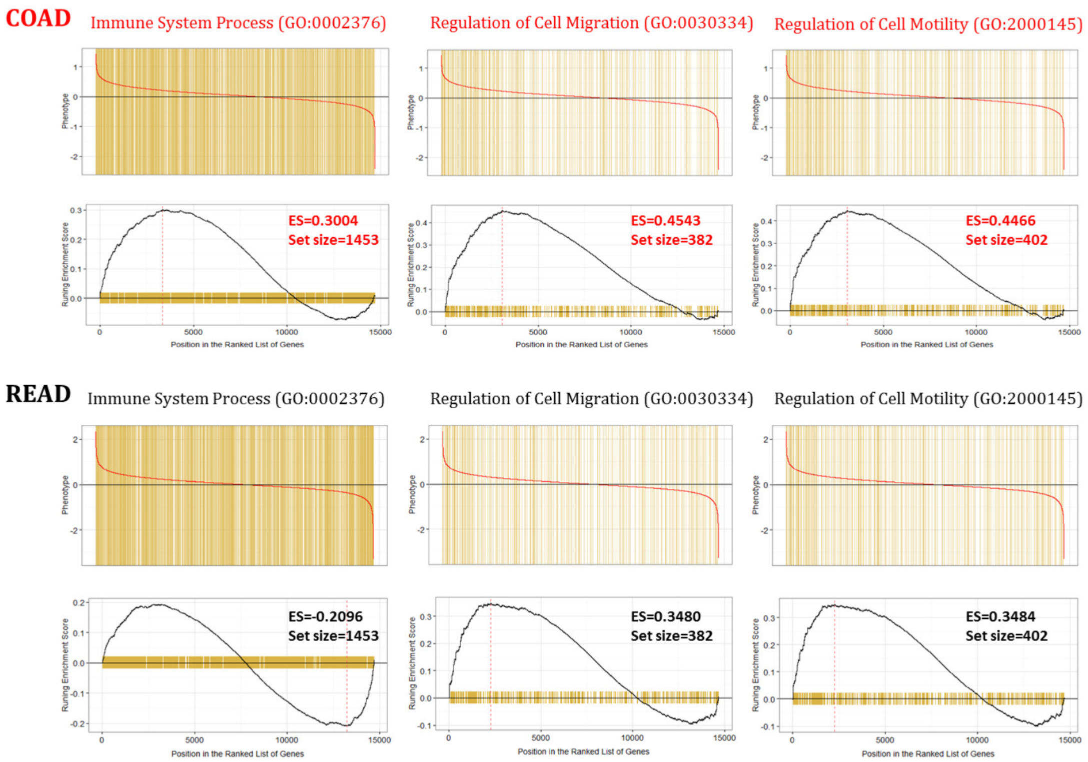

Supplementary Figure S3: Gene set enrichment analysis (GSEA) results of immune system processes, regulation of cell migration and regulation of cell motility on colon adenocarcinoma (COAD) and rectal adenocarcinoma (READ) expression datasets. The GSEA was performed based on gene ontology (GO) biological process (BP) terms, and the running sum enrichment score (ES) is shown. The BP term, "immune system process", was strongly positively correlated with genes that positively associated with *STIM1* overexpression signature in COADs (ES = 0.3004), but a reverse trend was observed in READs (ES = -0.2096). The BP terms, "regulation of cell migration" and "regulation of cell motility", were more strongly positively correlated with genes that positively associated with the *STIM1* overexpression signature in COADs (ES = 0.4543 and 0.4466, respectively) than READs (ES = 0.3480 and 0.3484, respectively).

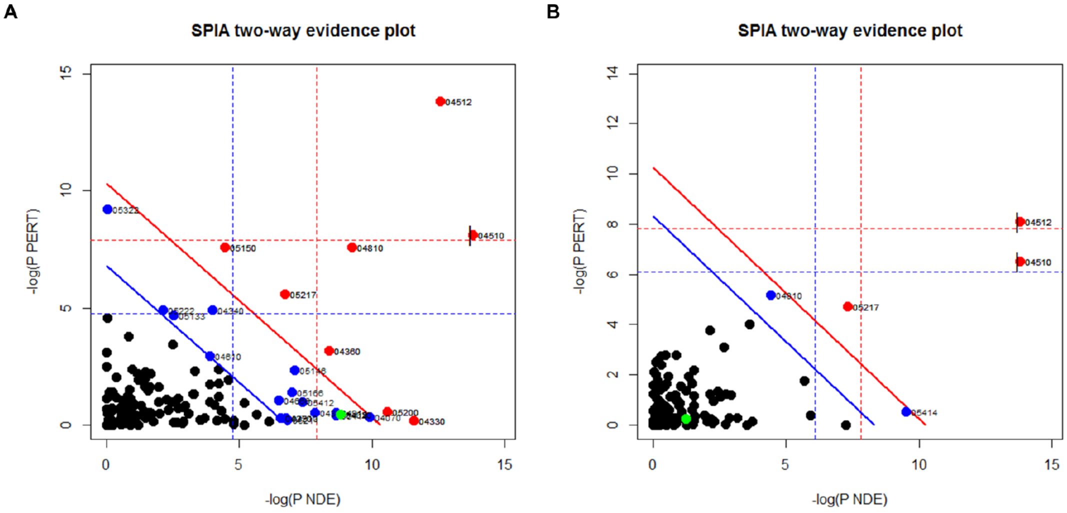

Supplementary Figure S4: Signaling pathway impact analysis results of RNA-sequencing expression data in A. COAD and B. READ patients. X-axis denoted the corresponding -log2 transformed overrepresentation *p*-value and the y-axis denoted the corresponding -log2 transformed perturbation *p*-value. Each KEGG pathway was plotted as circle dot based on calculated combined global *p*-value. The blue dots indicated the global *p*-value of corresponding pathways that reached FDR significant; while the red dots indicated the global *p*-value of corresponding pathways that reached Bonferroni significant. The green dot represented calcium signaling pathway (KEGG ID: hsa04020) in **A.** COADs and **B.** READs.

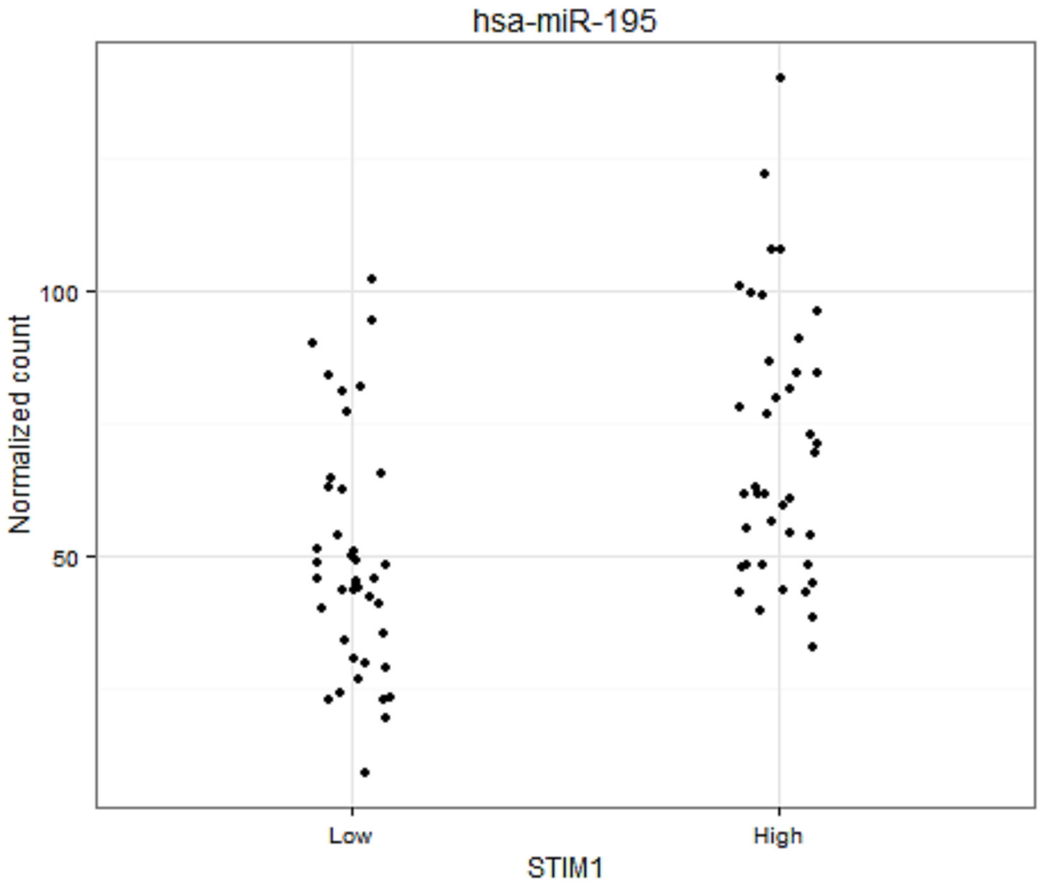

Supplementary Figure S5: Jitter plot showed the difference of normalized hsa-miR-195 count in *STIM1* overexpression group and *STIM1* low-expression group. The significance of differentially expression of hsa-miR-195 was confirmed by a generalized linear model under negative binomial distribution after controlled the mean-dependent variance. Besides, hsa-miR-195 was the only differentially expressed miRNA associated with COAD patients' survival profile.

A Prognostic predictor = 0.144×PANX1 + 0.167 × ETS1 + 0.138 × EXT1 + 0.153*C1QTNF6 + 0.162 × PCDH7 + 0.137 × GLIS3 + 0.148 × MARVELD1 + 0.139 × PPP2R3A + 0.160 × KIAA0247 + 0.154 × LRP12 + 0.056 × hsa-miR-195

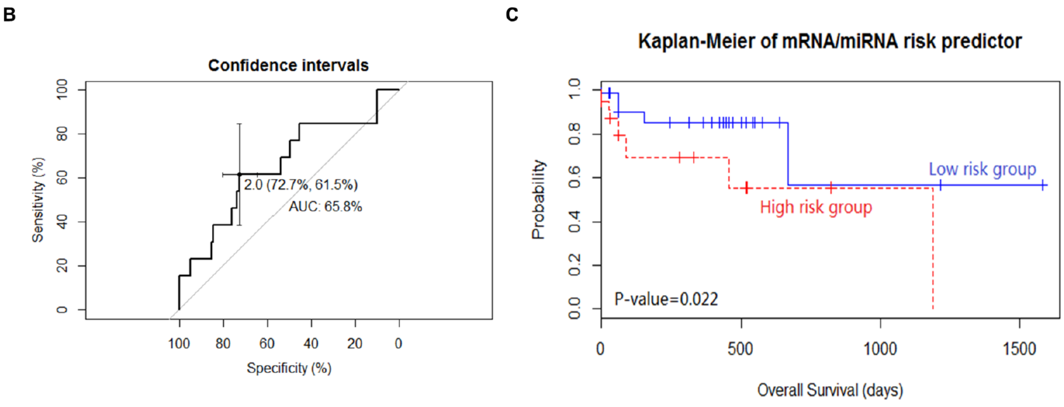

Supplementary Figure S6: Analysis of the prognostic mRNA/miRNA signatures identification in all 134 COAD patients and their performance in survival prediction. **A.** The equation of prognostic predictor calculated by PCA algorithm. **B.** ROC of prognostic predictor with 1-specificity as x-axis and sensitivity as y-axis. The best threshold (prognostic predictor = 2.0) and corresponding specificity and sensitivity (72.7% and 61.5%, respectively) were shown. Besides, the AUROC of 65.8% was shown. **C.** Kaplan-Meier curve for overall survival for dichotomized prognostic predictor. *P*-value calculated by Cox regression analysis. Data were from all available COAD patients without considering the *STIM1* expression status.

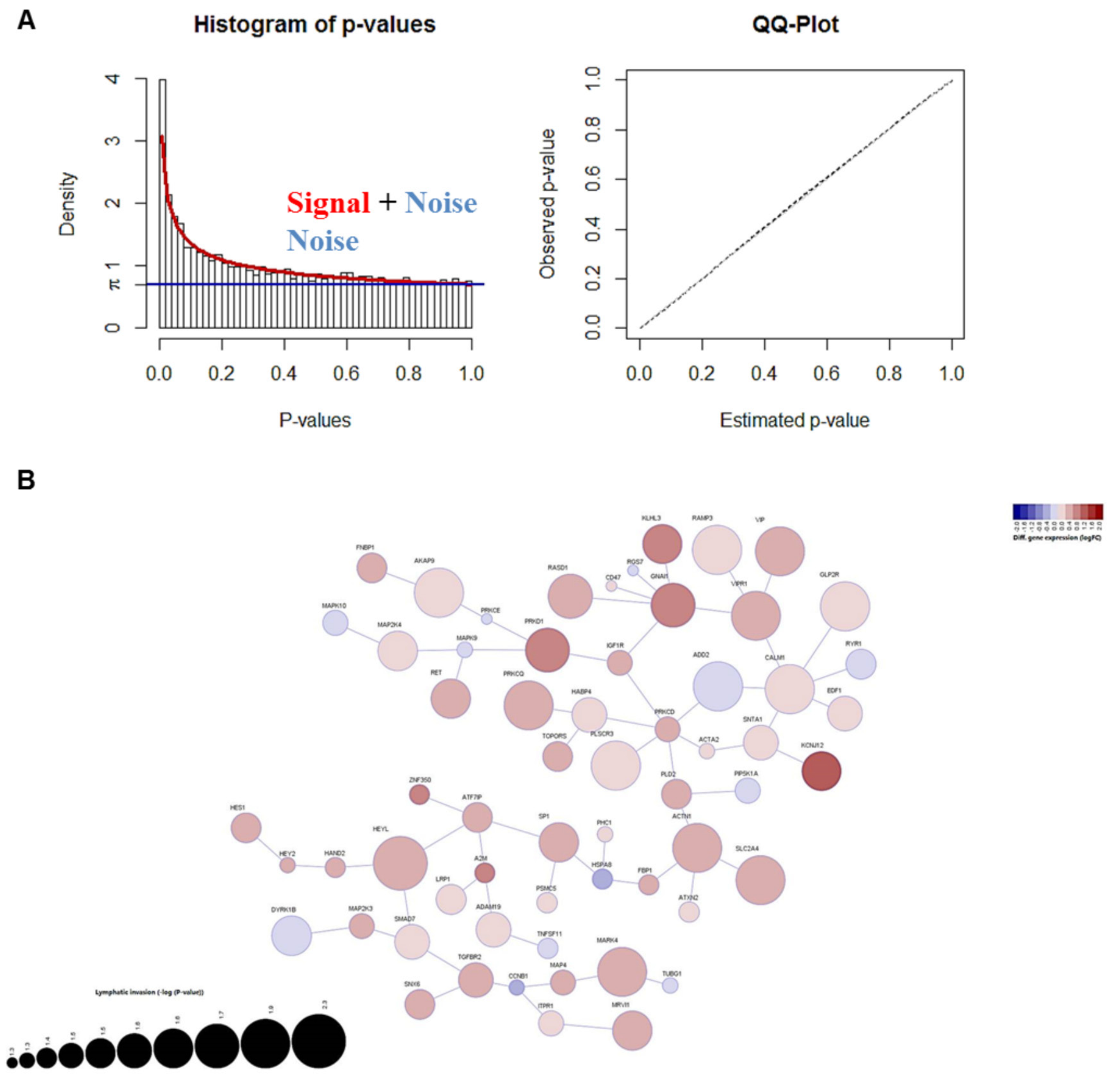

Supplementary Figure S7: Protein-protein interaction network analysis. A. In pathway analysis, DEGs *p*-value and lymphatic invasion related *p*-value were calculated. Two *p*-values of each signature were aggregated to become a second-order statistic to fit beta-uniform mixture model (BUM) to the distribution. The histogram (left diagram) showed the fitted BUM model of the *p*-values. Quantile-quantile plot (right diagram) revealed a nice fit of the BUM model. **B.** Protein-protein interaction network for differentially expressed genes in colonic adenocarcinomas (COADs) related to lymphatic invasion. The identified network module was correlated with the STIM1 overexpression profile and embodied the invasiveness ability of STIM1-associated signatures. The degrees of log2 multiples of change were mapped to node color, and correlations to lymphatic invasion (-log10 *p* value) were mapped to the node size. A larger size of a node indicates a higher association of the corresponding gene to the lymphatic invasion ability.

Supplementary Table S1A: Microarray profile of colorectal cancer patients

CRC Subtype	No. of samples	Up-regulated DEGs[a]	Down-regulated DEGs[b]
COAD	47	306	139
READ	22	0	0

Total genes: 14688. Significant threshold: DEGs with FDR value < 0.1.

[a]Up-regulated DEGs were defined by $\log_2$(multiple of change) > 0.

[b]Down-regulated DEGs were defined by $\log_2$(multiple of change) < 0.

Supplementary Table S1B: RNA sequencing profile in colorectal cancer patients

CRC Subtype	No. of samples	Up-regulated DEGs[a]	Down-regulated DEGs[b]
COAD	56	3482	517
READ	21	0	0

Total genes: 14727. Significant threshold: DEGs with FDR value < 0.1.
[a]Up-regulated DEGs were defined by $\log_2$(multiple of change) > 0.
[b]Down-regulated DEGs were defined by $\log_2$(multiple of change) < 0.

Supplementary Table S2: The co-expression patterns of transcripts mic profiles between COADs and READs in CRC patients.

Supplementary Table S3: Raw GSEA statistics of COAD and READ GO enrichment analysis

GO ID	GO Description	ES_{COAD}	P_{COAD}[a]	q_{COAD}[b]	ES_{READ}	P_{READ}	q_{READ}
GO:0030334	regulation of cell migration	0.454	< 0.001	< 0.001	0.348	< 0.001	< 0.001
GO:2000145	regulation of cell motility	0.447	< 0.001	< 0.001	0.348	< 0.001	< 0.001
GO:0002376	immune system process	0.300	< 0.001	< 0.001	-0.210	< 0.001	< 0.001

[a]*P*-value.
[b]*q*-value.

Supplementary Table S4: Difference of normalized enrichment score (NES) between COAD versus READ patients under gene set enrichment analysis of GO biological process (BP) terms

GO ID[a]	GO Description	Set Size[b]	NES_{COAD}[c]	NES_{READ}[d]	Diff.[e]
GO:0002376	immune system process	1453	0.155119	–1.27547	1.43059
GO:0030334	regulation of cell migration	382	0.825597	0.66896	0.15664
GO:2000145	regulation of cell motility	402	0.79181	0.670276	0.12153
GO:0016055	Wnt signaling pathway	222	0.698663	0.821884	–0.12322
GO:0022008	Neurogenesis	887	0.351283	0.475533	–0.12425
GO:0051239	regulation of multicellular organismal process	1417	0.346829	0.476334	–0.12950
GO:0006928	cellular component movement	1071	0.43209	0.562178	–0.13009
GO:0050794	regulation of cellular process	5187	0.177142	0.308401	–0.13126
GO:0048468	cell development	1168	0.310979	0.442651	–0.13167
GO:0034330	cell junction organization	168	0.722702	0.860334	–0.13763
GO:0050789	regulation of biological process	5483	0.161271	0.30126	–0.13999
GO:0001944	vasculature development	410	0.634379	0.774558	–0.14018
GO:0023052	Signaling	3385	0.248092	0.392425	–0.14433
GO:0044700	single organism signaling	3385	0.248092	0.392425	–0.14433
GO:0001568	blood vessel development	390	0.648622	0.794935	–0.14631
GO:0007154	cell communication	3427	0.243898	0.394059	–0.15016
GO:0007399	nervous system development	1284	0.340288	0.49098	–0.15069
GO:0048869	cellular developmental process	2090	0.261567	0.417902	–0.15634
GO:0048523	negative regulation of cellular process	2142	0.190856	0.351325	–0.16047
GO:0048519	negative regulation of biological process	2357	0.166984	0.328129	–0.16114
GO:0065007	biological regulation	5782	0.133422	0.300917	–0.16750
GO:0030154	cell differentiation	1999	0.243912	0.411688	–0.16778
GO:0022610	biological adhesion	701	0.526811	0.698457	–0.17165
GO:0007155	cell adhesion	698	0.523385	0.697138	–0.17375

(*Continued*)

GO ID[a]	GO Description	Set Size[b]	NES_{COAD}[c]	NES_{READ}[d]	Diff.[e]
GO:0048513	organ development	1802	0.202126	0.390504	-0.18838
GO:0007167	enzyme linked receptor protein signaling pathway	710	0.382922	0.581845	–0.19892
GO:0048856	anatomical structure development	2814	0.245752	0.46525	–0.21950
GO:0032502	developmental process	3121	0.224096	0.44479	–0.22069
GO:0044767	single-organism developmental process	3093	0.218239	0.440226	–0.22199
GO:0032501	multicellular organismal process	3727	0.174452	0.399227	–0.22477
GO:0007275	multicellular organismal development	2771	0.23331	0.460112	–0.22680
GO:0048731	system development	2457	0.261543	0.488454	–0.22691
GO:0044707	single-multicellular organism process	3618	0.168039	0.402298	–0.23426
GO:0048514	blood vessel morphogenesis	339	0.632324	0.869348	–0.23702
GO:0001525	Angiogenesis	278	0.674586	0.91202	–0.23743
GO:0007010	cytoskeleton organization	577	0.334771	0.582083	–0.24731
GO:0044699	single-organism process	7161	0.041424	0.334886	–0.29346
GO:0044763	single-organism cellular process	6549	0.020478	0.332282	–0.31180
GO:0007169	transmembrane receptor protein tyrosine kinase signaling pathway	518	0.366556	0.678716	–0.31216
GO:0030036	actin cytoskeleton organization	333	0.547441	0.865664	–0.31822
GO:0030029	actin filament-based process	369	0.467179	0.862629	–0.39545
GO:0016071	mRNA metabolic process	386	–2.3325	–1.71178	–0.62072
GO:0006412	Translation	327	–2.50983	–1.77429	–0.73554
GO:0032984	macromolecular complex disassembly	151	–2.81909	–2.07124	–0.74785
GO:0072657	protein localization to membrane	255	–2.44453	–1.64718	–0.79735

[a]GO: gene ontology.
[b]The number of genes involved in a GO BP gene set.
[c]Normalized enrichment scores of colorectal adenocarcinoma related ranked list.
[d]Normalized enrichment scores of rectal adenocarcinoma related ranked-list.
[e]Difference of NES was calculated by COAD NES—READ NES. Criteria: Diff. of NES > 0.12; gene set size > 150; FDR value $< 5 \times 10^{-5}$ in both COAD and READ ranked list.

Supplementary Table S5A: Signaling pathway impact analysis (SPIA) results in COAD patients

No.	Name	KEGG ID	Set Size[a]	NDE[b]	P_{NDE}[c]	$PERT_{Total}$[d]	P_{PERT}	P_{Global}[e]	$P_{Global\ FDR}$	$P_{Global\ FWER}$[f]	Status[g]
1	Focal adhesion	4510	166	83	1.77×10^{-09}	38.69	3.00×10^{-04}	1.56×10^{-11}	2.13×10^{-09}	2.13×10^{-09}	Activated
2	ECM-receptor interaction	4512	65	36	3.50×10^{-06}	13.24	5.00×10^{-07}	4.92×10^{-11}	3.37×10^{-09}	6.74×10^{-09}	Activated
3	Regulation of actin cytoskeleton	4810	185	76	9.52×10^{-05}	32.88	5.00×10^{-04}	8.51×10^{-07}	3.88×10^{-05}	0.000117	Activated
4	Basal cell carcinoma	5217	28	16	0.0012	10.59	0.0038	6.07×10^{-05}	0.002078	0.00831	Activated
5	Staphylococcus aureus infection	5150	50	22	0.011779	15.54	5.00×10^{-04}	7.68×10^{-05}	0.002105	0.010523	Activated
6	Notch signaling pathway	4330	43	26	9.39×10^{-06}	-1.10	0.8375	0.0001	0.002289	0.013737	Inhibited
7	Axon guidance	4360	89	41	0.00023	8.27	0.0417	0.00012	0.002353	0.016474	Activated
8	Pathways in cancer	5200	252	101	2.53×10^{-05}	8.02	0.5724	0.000176	0.003012	0.024097	Activated
9	Phosphatidylinositol signaling system	4070	79	39	5.02×10^{-05}	0.30	0.7063	0.000399	0.006071	0.054636	Activated
10	Lysosome	4142	109	47	0.000551	0.00	NA	0.000551	0.007554	0.075543	Inhibited
11	Amoebiasis	5146	88	39	0.000834	3.78	0.0976	0.000848	0.010407	0.116168	Activated
12	Calcium signaling pathway	4020	158	66	0.00015	2.45	0.6314	0.000971	0.010407	0.133015	Activated
13	Systemic lupus erythematosus	5322	120	24	0.984455	7.58	1.00×10^{-04}	0.001007	0.010407	0.137919	Activated
14	Progesterone-mediated oocyte maturation	4914	80	38	0.000176	-1.96	0.594	0.001063	0.010407	0.145692	Inhibited
15	Dilated cardiomyopathy	5414	72	35	0.000177	-0.51	0.674	0.0012	0.010957	0.164358	Inhibited
16	Hedgehog signaling pathway	4340	32	15	0.018415	8.88	0.0074	0.001349	0.011553	0.184843	Activated
17	Osteoclast differentiation	4380	102	45	0.000391	2.56	0.5809	0.002134	0.015677	0.2924	Activated
18	HTLV-I infection	5166	197	76	0.000917	5.20	0.252	0.002166	0.015677	0.296756	Activated
19	Arrhythmogenic right ventricular cardiomyopathy (ARVC)	5412	65	31	0.000619	0.51	0.3748	0.002174	0.015677	0.297863	Activated
20	Fc gamma R-mediated phagocytosis	4666	79	35	0.001516	4.71	0.3483	0.004513	0.030914	0.618284	Activated
21	Pertussis	5133	62	23	0.078973	9.34	0.0093	0.006035	0.039368	0.826728	Activated
22	Insulin signaling pathway	4910	115	48	0.001149	-3.02	0.7313	0.006792	0.040802	0.930515	Inhibited
23	Small cell lung cancer	5222	71	25	0.117307	13.38	0.0074	0.006987	0.040802	0.957262	Activated
24	Renal cell carcinoma	5211	56	27	0.001106	0.84	0.8054	0.007148	0.040802	0.979245	Activated
25	Vascular smooth muscle contraction	4270	113	47	0.001405	2.02	0.7481	0.008258	0.045135	1	Activated
26	Complement and coagulation cascades	4610	58	24	0.020484	14.09	0.0535	0.008566	0.045135	1	Activated
27	Prostate cancer	5215	80	32	0.014523	10.26	0.0914	0.010121	0.051353	1	Activated

(*Continued*)

No.	Name	KEGG ID	Set Size[a]	NDE[b]	P_{NDE}[c]	$PERT_{Total}$[d]	P_{PERT}	P_{Global}[e]	$P_{Global\ FDR}$	$P_{Global\ FWER}$[f]	Status[g]
28	Leukocyte transendothelial migration	4670	102	40	0.010028	9.56	0.1477	0.011131	0.054462	1	Activated
29	Acute myeloid leukemia	5221	42	21	0.002184	0.40	0.8682	0.013782	0.06511	1	Activated
30	Gap junction	4540	67	29	0.005559	5.08	0.3885	0.015415	0.068472	1	Activated
31	B cell receptor signaling pathway	4662	68	30	0.003446	-1.95	0.6305	0.015494	0.068472	1	Inhibited
32	Chagas disease (American trypanosomiasis)	5142	86	34	0.014607	6.87	0.1706	0.017431	0.074626	1	Activated
33	TGF-beta signaling pathway	4350	53	20	0.082964	–7.99	0.0326	0.018697	0.077619	1	Inhibited
34	Type II diabetes mellitus	4930	40	17	0.035993	–5.58	0.0987	0.023589	0.093478	1	Inhibited
35	GnRH signaling pathway	4912	97	37	0.020679	10.02	0.1743	0.023881	0.093478	1	Activated

[a]Number of genes on corresponding pathway.
[b]Number of differentially enriched genes per pathway.
[c]Probability under hypergeometric model.
[d]The observed total perturbation accumulation in the pathway.
[e]Combined *p*-value calculated from P_{NDE} and P_{PERT} by Fisher's combined method.
[f]Bonferroni-adjusted global *p*-values.
[g]Direction in which the pathway is perturbed.
Significant threshold: $P_{Global\ FDR} < 0.1$.

Supplementary Table S5B: Signaling pathway impact analysis (SPIA) results in READ patients

No.	Name	KEGG ID	Set Size[a]	NDE[b]	P_{NDE}[c]	$PERT_{Total}$[d]	P_{PERT}	P_{Global}[e]	$P_{Global\ FDR}$	$P_{Global\ FWER}$[f]	Status[g]
1	ECM-receptor interaction	4512	65	26	3.11×10^{-11}	8.67	3.00×10^{-04}	3.11×10^{-13}	3.95×10^{-11}	3.95×10^{-11}	Activated
2	Focal adhesion	4510	166	37	3.50×10^{-07}	28.69	0.0015	1.17×10^{-08}	7.46×10^{-07}	1.49×10^{-06}	Activated
3	Basal cell carcinoma	5217	28	9	0.000668	9.75	0.0089	7.75×10^{-05}	0.00328	0.009841	Activated
4	Dilated cardiomyopathy	5414	72	18	7.34×10^{-05}	-0.71	0.5973	0.000484	0.015361	0.061443	Inhibited
5	Regulation of actin cytoskeleton	4810	185	27	0.011931	19.26	0.0057	0.000721	0.018304	0.091518	Activated
6	Lysosome	4142	109	20	0.002263	0.00	NA	0.002263	0.047898	0.287389	Inhibited
7	Shigellosis	5131	47	9	0.026771	7.52	0.0182	0.004203	0.076258	0.533809	Activated
8	Pathways in cancer	5200	252	37	0.003339	15.40	0.1745	0.004922	0.078133	0.625064	Activated
9	Arrhythmogenic right ventricular cardiomyopathy (ARVC)	5412	65	15	0.000721	0.00	1	0.005934	0.083739	0.75365	Inhibited

Significant threshold: $P_{Global\ FDR} < 0.1$.
[a]Number of genes on corresponding pathway.
[b]Number of differentially enriched genes per pathway.
[c]Probability under hypergeometric model.
[d]The observed total perturbation accumulation in the pathway.
[e]Combined *p*-value calculated from P_{NDE} and P_{PERT} by Fisher's combined method.
[f]Bonferroni-adjusted global *p*-values.
[g]Direction in which the pathway is perturbed.

Supplementary Table S6: Calcium signaling pathway (KEGG ID: hsa04020) in COADs and READs

hsa04020	Set Size[a]	NDE[b]	P_{NDE}[c]	$PERT_{Total}$[d]	P_{PERT}	P_{Global}[e]	$P_{Global\ FDR}$	$P_{Global\ FWER}$[f]	Status[g]
COAD	158	66	0.00015	2.45	0.6314	0.000971	0.010407	0.133015	Activated
READ	158	17	0.29569	-0.90	0.7922	0.574226	1	1	Inhibited

[a]Number of analysed genes in calcium signaling pathway (KEGG ID: hsa04020).
[b]Number of differentially enriched genes per pathway.
[c]Probability under hypergeometric model.
[d]The observed total perturbation accumulation in the pathway.
[e]Combined *p*-value calculated from P_{NDE} and P_{PERT} by Fisher's combined method.
[f]Bonferroni-adjusted global *p*-values.
[g]Direction in which the pathway is perturbed.

Supplementary Table S7A: Differential expressed analysis result of miRNA sequencing data in COAD patients

miRNA	log_2(FC)[a]	Std Err.[b]	Stat.[c]	*P*-value[d]	P_{adj}[e]
hsa-miR-130b	–0.56206	0.118827	–4.73008	2.24E–06	0.00059
hsa-miR-17	–0.64906	0.16216	–4.00259	6.27E–05	0.00731
hsa-miR-195	0.507507	0.131315	3.864796	0.000111	0.00731
hsa-miR-497	0.536734	0.138552	3.873893	0.000107	0.00731
hsa-miR-20a	–0.62971	0.166817	–3.77484	0.00016	0.008422
hsa-miR-143	0.770307	0.216941	3.550774	0.000384	0.01615
hsa-miR-25	–0.39632	0.112556	–3.52104	0.00043	0.01615
hsa-miR-24-1	0.419278	0.125022	3.353636	0.000798	0.021012
hsa-miR-576	–0.57832	0.172469	–3.35317	0.000799	0.021012
hsa-miR-93	–0.52813	0.154956	–3.40828	0.000654	0.021012
hsa-miR-29c	0.629664	0.193381	3.256072	0.00113	0.027009
hsa-miR-574	0.437515	0.139895	3.127462	0.001763	0.038644
hsa-miR-942	–0.53318	0.176052	–3.02853	0.002457	0.049716
hsa-miR-483	–1.20507	0.408667	–2.94878	0.00319	0.059932
hsa-miR-18a	–0.46658	0.162697	–2.86777	0.004134	0.067948
hsa-miR-200c	–0.44832	0.155472	–2.88363	0.003931	0.067948
hsa-miR-26a-2	0.34237	0.121169	2.825552	0.00472	0.071499
hsa-miR-30a	0.357755	0.127136	2.813964	0.004893	0.071499
hsa-miR-584	–0.54414	0.19714	–2.76018	0.005777	0.075967
hsa-miR-92a-2	–0.49969	0.18048	–2.76867	0.005629	0.075967
hsa-let-7e	0.395461	0.148856	2.65667	0.007892	0.08648
hsa-let-7f-1	–0.35236	0.130762	–2.69466	0.007046	0.08648
hsa-miR-106b	–0.25145	0.094526	–2.66009	0.007812	0.08648
hsa-miR-150	0.521328	0.195741	2.663362	0.007736	0.08648
hsa-miR-10a	–0.51203	0.19449	–2.63268	0.008471	0.08912
hsa-miR-616	–0.47895	0.183312	–2.61278	0.008981	0.090846

[a]Fold change in log_2 transformation.
[b]Standard error of the corresponding log_2 fold change value.
[c]Statistics of generalized linear model.
[d]Wald test *p*-value.
[e]FDR-adjusted *p*-value.

Supplementary Table S7B: Differential expressed analysis result of miRNA sequencing data in READ patients

miRNA	log_2(FC)[a]	Std Err.[b]	Stat.[c]	*P*-value[d]	P_{adj}[e]
hsa-miR-1978	–1.13423	0.301364	-3.76364	0.000167	0.055094
hsa-miR-203	–0.99652	0.290277	-3.43299	0.000597	0.098199

[a]Fold change in log_2 transformation.
[b]Standard error of the corresponding log_2 fold change value.
[c]Statistics of generalized linear model.
[d]Wald test *p*-value.
[e]FDR-adjusted *p*-value.

Supplementary Table S7C: Differentially regulated miRNAs that had been reported to associated with CRC tumor initiation and progression by colorectal cancer subtypes with FDR-adjusted *p*-value < 0.1

miRNA	CRC Association[a]	Ref.[b]
hsa-miR-10a	Maintenance of the stemness in CD44(+) HCT-15 and HCT-116 human colon cancer cells	[1]
hsa-miR-130b	Suppresses migration and invasion of colorectal cancer cells through downregulation of integrin β1	[2]
hsa-miR-143	Regulate IGF1R to suppress cell proliferation	[3]
hsa-miR-150	Targeting c-Myb to regulate tumourigenesis and progression	[4]
hsa-miR-17	Inhibits TGF-β signaling	[5]
hsa-miR-18a	Inhibits CDC42 and CCND1 to exert growth-suppression effect	[6]
hsa-miR-195	Suppress CARMA3 protein expression and correlate with lymph node metastasis and poor prognosis	[7]
hsa-miR-200c	Regulates Sox2 expression through a feedback loop and is associated with CRC stemness, growth, and metastasis.	[8]
hsa-miR-25	Negatively regulate Angiopoietin-like protein 2 contributes to the malignant progression	[9]
hsa-miR-29c	mediates epithelial-to-mesenchymal transition via PTP4A and GNA13 regulation of β-catenin signaling	[10]
hsa-miR-30a	Suppress colon cancer cell growth through inhibition of IRS2	[11]
hsa-miR-497	Targets insulin-like growth factor 1 receptor and has a tumour suppressive role	[12]
hsa-miR-93	Suppress colorectal cancer development via Wnt/β-catenin pathway downregulating	[13]
hsa-miR-203	Related to the proliferation and invasion of cancers.	[14]

[a]The miRNAs function (promoted or inhibited) on CRC tumors.
[b]References.

Supplementary Table S8: Results of multistep clinical subclasses based survival analysis

mRNA/miRNA	*P*-value					HR (95% CI)[a]
	Tumor stage	Lymph node spread	Distant metastasis	Vascular invasion	Lymphatic invasion	
PANX1	0.0067**	0.0024**	0.0063**	0.0253*	0.0135*	15.49 (14.49–16.49)
ETS1	0.0087**	0.0103*	0.0083**	0.4292	0.0213*	4.17 (3.62–4.73)
EXT1	0.0088**	0.0040**	0.0087**	0.0124*	0.0155*	17.94 (16.96–18.92)
C1QTNF6	0.0105*	0.0101*	0.0118*	0.1139	0.0307*	8.46 (7.63–9.30)
PCDH7	0.0205*	0.0110*	0.0152*	0.0710	0.0174*	8.82 (7.97–9.68)
GLIS3	0.0316*	0.0139*	0.0182*	0.0970	0.0532	2.15 (1.81–2.49)
MARVELD1	0.0351*	0.0387*	0.0321*	0.2811	0.0711	8.27 (7.32–9.23)
PPP2R3A	0.0378*	0.0205*	0.0289*	0.0638	0.0520	4.74 (4.04–5.44)
KIAA0247	0.0477*	0.0560	0.0454*	0.1329	0.1555	5.24 (4.39–6.09)
LRP12	0.0813	0.0321*	0.0378*	0.0662	0.0410*	5.50 (4.69–6.32)
hsa-mir-195	0.0525	0.0399*	0.0368*	0.2258	0.0679	1.000773 (1.000424–1.001122)

Survival analysis was conducted to identify significant prognostic mRNA/miRNA signature. Cox-proportional hazard model was fitted to calculate corresponding hazard ratios and *p*-value.
[a]Hazard ratios (95% Confidence intervals) was calculated by univariate survival analysis.
[*]*P*-value < 0.05.
[**]*P*-value < 0.01.

SUPPLEMENTARY NOTE

Interactome Analysis to Identify Differentially Expressed Modules in COADs Simultaneously Associated with Lymphatic Invasion.

To determine the aberrant subgraph responsible for the functional features of *STIM1* overexpression, we conducted a network analysis using HPRD molecular network interaction information. In brief, genes with moderated *t*-test *p* value of < 0.05 in COADs were further tested for the correlation between the expression value and lymphatic invasion status (present or absent). Then, only genes with *p* values of < 0.05 were selected for an interactome analysis. DEG *p* values and lymphatic-invasion related *p* values were further aggregated to fit a beta-uniform distribution (Supplementary Figure S7A). Next, all genes in the network were scored (FDR threshold of < 10^{-5}), and finally a maximum scoring network was identified by a heuristic algorithm [11].

The protein-protein interaction network from Human Protein Reference Database (HPRD, April 2011), a manually curated human protein database [12], was incorporated with DEGs and clinical lymphatic invasion information to identify significant network modules. The resultant subnetwork is displayed in Supplementary Figure S7B. As shown in this figure, most of the genes associated with lymphatic invasion were upregulated in the *STIM1* overexpression group, indicating that activation of transcription of *STIM1* and *STIM1*-associated signatures could increase clinical aspects of tumor aggressiveness. Moreover, most of these genes are known to be cancer-associated or to participate in the invasion-metastasis cascade.The co-expression patterns of transcripts mic profiles between COADs and READs in CRC patients.

REFERENCES

1. Ju, S.Y., S.H. Chiou, Y. Su. Maintenance of the stemness in CD44(+) HCT-15 and HCT-116 human colon cancer cells requires miR-203 suppression. Stem Cell Res. 2014; 12:86–100.
2. Zhao, Y., G. Miao, Y. Li, T. Isaji, J. Gu, J. Li, R. Qi. MicroRNA- 130b suppresses migration and invasion of colorectal cancer cells through downregulation of integrin beta1[corrected]. PLoS One. 2014; 9:e87938.
3. Su, J., H. Liang, W. Yao, N. Wang, S. Zhang, X. Yan, H. Feng, W. Pang, Y. Wang, X. Wang, Z. Fu, Y. Liu, C. Zhao, et al. MiR-143 and MiR-145 Regulate IGF1R to Suppress Cell Proliferation in Colorectal Cancer. PLoS One. 2014; 9:e114420.

4. Feng, J., Y. Yang, P. Zhang, F. Wang, Y. Ma, H. Qin, Y. Wang. miR-150 functions as a tumour suppressor in human colorectal cancer by targeting c-Myb. J Cell Mol Med. 2014; 18:2125–34.
5. Dews, M., G.S. Tan, S. Hultine, P. Raman, J. Choi, E.K. Duperret, J. Lawler, A. Bass, A. Thomas-Tikhonenko. Masking epistasis between MYC and TGF-beta pathways in antiangiogenesis-mediated colon cancer suppression. J Natl Cancer Inst. 2014; 106:dju043.
6. Humphreys, K.J., R.A. McKinnon, M.Z. Michael. miR-18a inhibits CDC42 and plays a tumour suppressor role in colorectal cancer cells. PLoS One. 2014; 9:e112288.
7. Wang, L., L. Qian, X. Li, J. Yan. MicroRNA-195 inhibits colorectal cancer cell proliferation, colony-formation and invasion through targeting CARMA3. Mol Med Rep. 2014; 10:473–8.
8. Lu, Y.X., L. Yuan, X.L. Xue, M. Zhou, Y. Liu, C. Zhang, J.P. Li, L. Zheng, M. Hong, X.N. Li. Regulation of colorectal carcinoma stemness, growth, and metastasis by an miR-200c-Sox2-negative feedback loop mechanism. Clin Cancer Res. 2014; 20:2631–42.
9. Zhou, J., J. Wang, S. Wu, S. Zhu, S. Wang, H. Zhou, X. Tian, N. Tang, S. Nie. Angiopoietin-like protein 2 negatively regulated by microRNA-25 contributes to the malignant progression of colorectal cancer. Int J Mol Med. 2014; 34:1286–92.
10. Zhang, J.X., S.J. Mai, X.X. Huang, F.W. Wang, Y.J. Liao, M.C. Lin, H.F. Kung, Y.X. Zeng, D. Xie. MiR-29c mediates epithelial-to-mesenchymal transition in human colorectal carcinoma metastasis via PTP4A and GNA13 regulation of beta-catenin signaling. Ann Oncol. 2014; 25:2196–204.
11. Zhang, Q., Q. Tang, D. Qin, L. Yu, R. Huang, G. Lv, Z. Zou, X.C. Jiang, C. Zou, W. Liu, J. Luo, Z. Zhao, S. Muhammad, et al. Role of miR-30a targeting Insulin Receptor Substrate 2 in colorectal tumorigenesis. Mol Cell Biol. 2015; .
12. Guo, S.T., C.C. Jiang, G.P. Wang, Y.P. Li, C.Y. Wang, X.Y. Guo, R.H. Yang, Y. Feng, F.H. Wang, H.Y. Tseng, R.F. Thorne, L. Jin, X.D. Zhang. MicroRNA-497 targets insulin-like growth factor 1 receptor and has a tumour suppressive role in human colorectal cancer. Oncogene. 2013; 32:1910–20.
13. Tang, Q., Z. Zou, C. Zou, Q. Zhang, R. Huang, X. Guan, Q. Li, Z. Han, D. Wang, H. Wei, X. Gao, X. Wang. MicroRNA-93 suppress colorectal cancer development via Wnt/beta-catenin pathway downregulating. Tumour Biol. 2014; .
14. Chiang, Y., Y. Song, Z. Wang, Y. Chen, Z. Yue, H. Xu, C. Xing, Z. Liu. Aberrant expression of miR-203 and its clinical significance in gastric and colorectal cancers. J Gastrointest Surg. 2011; 15:63–70.
